# Kinetic Insights into Cholera Toxin B‑Biomimetic Glycan Interactions

**DOI:** 10.1021/acs.biomac.5c02759

**Published:** 2026-04-07

**Authors:** Kun Lin Hsieh, Navanjalee T. Panagoda, Jermaine L. Jenkins, Nicole S. Sampson

**Affiliations:** † Department of Chemistry, 603074 Stony Brook University, Stony Brook, New York 11794-3400, United States; ‡ Department of Chemistry, University of Rochester, Rochester, New York 14627-0216, United States; § Department of Biochemistry and Biophysics, 12299 University of Rochester, Rochester, New York 14642-0001, United States

## Abstract

Cholera is a diarrheal disease caused by *Vibrio cholerae*, which secretes cholera enterotoxin (CTX) in the small intestinal epithelium. The pentameric B subunit (CTB) of CTX binds to glycans on the cellular surface through a primary binding site for GM_1_ glycosphingolipid or galactose. However, GM_1_ is undetectable in the human SI, and fucose is a key alternative ligand. Previously, we developed linear norbornenyl glycopolymers that block CTB binding by forming CTB–glycopolymer aggregates. Here, we evaluate CTB–glycopolymer binding kinetics under flow conditions using surface plasmon resonance. A copolymer randomly displaying galactose and fucose formed stable complexes with nanomolar avidity, driven primarily by slow dissociation, even from low CTB density surfaces. In contrast, an equimolar mixture of homopolymers exhibited similar binding avidity and comparable inhibitory efficacy but did not display slow dissociation. These findings underscore the importance of codisplaying galactose and fucose on a single polymer backbone for stable complex formation and for developing clinically useful therapies.

## Graphical abstract

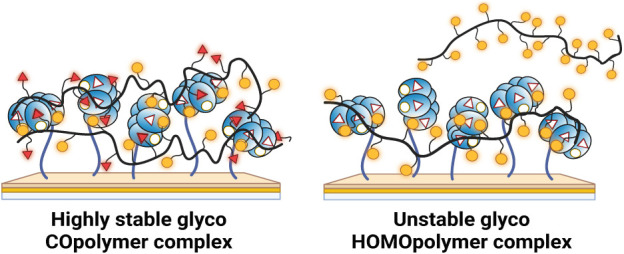

Enterotoxins are a major cause of bacterial cytotoxicity, targeting glycan receptors on intestinal epithelial cells and leading to diarrhea, dehydration, and severe illnesses such as hemorrhagic colitis.^1^ Among enterotoxin-secreting bacteria, cholera toxin (CTX), an AB_5_ lectin family member that is secreted by*Vibrio cholerae*, induces severe dehydration, diarrhea, and vomiting, particularly in infants and young children under five, due to their underdeveloped gastrointestinal immune systems.^2,3^ CTX is secreted after *V. cholerae* colonizes the small intestine, where it intoxicates the host by targeting specific glycan receptors on small intestinal epithelial cells (SI-ECs). The homopentameric B subunit (CTB) binds to these receptors, triggering endocytosis and retrograde transport to the endoplasmic reticulum, where the A subunit (CTA) dissociates.^4^ Once in the cytosol, CTA activates the G protein Gsα, promoting cyclic AMP (cAMP) production. The resulting increase in intracellular cAMP levels leads to excessive ion secretion, creating an osmotic imbalance that causes significant fluid loss, manifesting as life-threatening dehydration and diarrhea.^5^

The mortality rate for untreated cholera can reach 20–50%,^2^ underscoring the need for immediate access to medical treatment.^6^ Simultaneously, the global effort to eliminate cholera is hindered by a widespread lack of access to clean water, highlighting the importance of sustainable water infrastructure alongside clinical interventions. Current control strategies for cholera include antibiotics, oral vaccines, and oral rehydration solution (ORS) for symptoms ranging from mild to severe.^3^ Although ORS has highly reduced mortality, the solution does not provide therapeutic effects.^7^ In addition, the misuse and overuse of antibiotics have contributed to antibiotic resistance, reducing drug efficacy.^8^ Furthermore, oral cholera vaccines—including killed and live-attenuated versions—are available, but they provide only 65–70% protection, are relatively short-lived, and are highly age-dependent with lower efficacy among children in endemic regions.^6,9^ The challenge is especially critical for high-risk patients such as infants, and in endemic countries, a harmless, low-cost, and readily available therapy is urgently needed.

Human milk oligosaccharides (HMOs), a natural complex of glycans found in human breast milk, offer a promising protective role in infant intestinal health and development.^10^ Acidic HMOs contain saccharide residues that show similarities with the GM_1_ structure,^11^ as well as fucosylated structures such as 2′-FL, which can be recognized by CTB.^12,13^ However, HMO ligands have low binding affinities for enterotoxins even at high HMO concentrations.^14^

Motivated by natural products, glycomimetic inhibitors have been designed with diverse structures and compositions to neutralize CTX toxicity by blocking CTB binding to host epithelial cell receptors.^15,16^ Their structures include small molecules,^17–19^ bivalent,^20,21^ pentameric,^22–25^ and multivalent inhibitors with over 5 binding ligands.^26–28^ The multivalent designs are inspired by the pentameric structure of CTB (Figure 1B), which features two distinct glycan-binding sites on each monomer, as revealed in the crystal structures. These binding sites—specific for GM_1_/galactose and fucose residues—exhibit high and low affinities, respectively, and are both key targets to block CTX intoxication. Targeting multiple GM_1_-binding sites simultaneously has been a promising strategy for designing potent inhibitors.^29^ However, no synthetic inhibitors that exploit the C5 symmetry of CTB’s galactose-binding sites to engage multiple sites simultaneously have demonstrated sufficient efficacy in blocking intoxication for therapeutic applications.^22,30,31^ Although dual-ligand systems have been demonstrated to improve inhibition of CTB binding,^12,32,33^ it remains unclear whether the combination of galactose and fucose will form complexes of CTB–glycopolymer that are sufficiently kinetically stable for in vivo use. Resolving this question is key to developing more effective glycopolymer-based inhibitors of cholera toxin.

**Figure 1:**
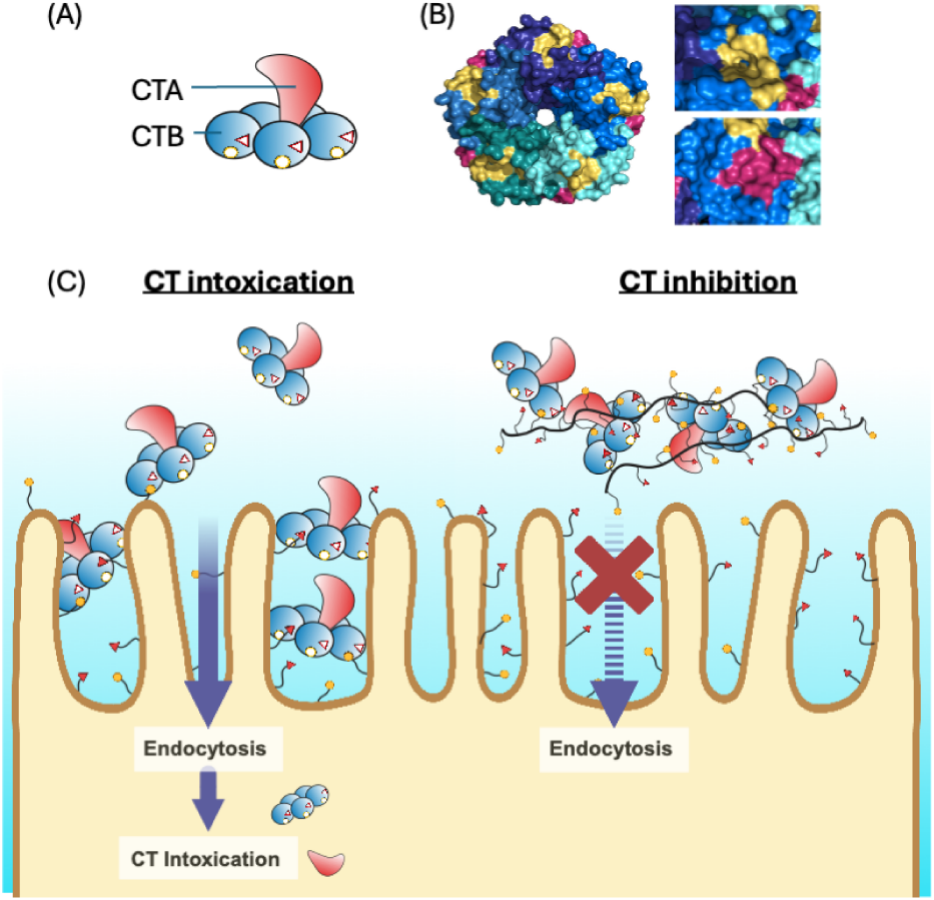
Cholera toxin tertiary structure and mechanism of cholera intoxication.^1^ (A) A cartoon representation of CT: cholera toxin A subunit (CTA, red) with a homopentameric CTB structure (blue) with open triangles and circles representing unoccupied binding sites for fucose and galactose, respectively. (B) Homopentameric CTB structure viewed from the bottom, highlighting the two binding sites: the canonical (yellow, GM_1_/galactose-binding site) and the noncanonical (red, fucose-binding site). Protein structures are based on PDB entries pdb_00006hmw (overall structure) and pdb_00006hmy (fucose-binding region) with close-up views of the noncanonical (red) and canonical (yellow) binding cavities. (C) CTB binds to glycan receptors on the epithelial cell surface, leading to CT intoxication (left), while glycopolymer blocks CTB binding to the cell surface by forming CTB–glycopolymer aggregates (right). Protein structures are based on PDB entries pdb_00006hmw (overall structure) and pdb_00006hmy (fucose-binding region).

Our previous studies have shown that copolymers containing both galactose and fucose, designed to take advantage of both the canonical and noncanonical sites, exhibit nanomolar inhibition efficacy blocking both binding to and intoxication of human enteroids.^33,34^ Additionally, we demonstrated that strong inhibition correlates with the formation of large CTB–copolymer aggregates (Figure 1C).^34^ These large aggregates are proposed to result from the facial directionality of the CTB structure, where the distinct binding sites are located on different faces of the pentamer (Figure 1), allowing simultaneous engagement of both sites and enhancing inhibition efficacy to the nanomolar scale.^34^ However, the dynamic interactions among CTB, glycopolymers, and their aggregate formation remain poorly understood.

In this study, we evaluated the binding kinetics of CTB–glycopolymer interactions using an open-system measurement to better mimic physiological conditions in the human body. An open system can reflect the shift in drug concentration over time. Therefore, the drug–target binding kinetics reveal the dynamic interaction under nonequilibrium conditions. We hypothesized that a random copolymer displaying both galactose and fucose assembles and binds tightly to CTB by forming a stable CTB–glycopolymer complex that prevents the release of the captured CTB during intestinal transit (Figure 1C). To assess the therapeutic potential of these copolymers, we analyzed CTB–glycopolymer binding kinetics by using a surface plasmon resonance (SPR) flow system. We compared the binding and dissociation rates of CTB–glycopolymers, including different lengths of random copolymers, homopolymer mixtures, and homopolymers with different ligands. Our results demonstrate that a random copolymer displaying both galactose and fucose forms a highly stable complex with the anchored CTB even after the dissociation phase, distinguishing it from the other polymers tested. In contrast, homopolymers underwent fast and reversible binding. All the findings fit a multivalent binding model, suggesting that multiple CTB–glycopolymer interactions occur simultaneously.

## Materials and Methods

### Materials

Materials were purchased from Sigma-Aldrich, Fisher Scientific, VWR, Santa Cruz Biotechnology, Thermo Fisher Scientific, Enzo Life Sciences, and Cytiva and used as received unless otherwise specified. Tetrahydrofuran (THF, Fisher Scientific, 99.8%), methanol (MeOH, Sigma-Aldrich, ≥99.8%), dimethylformamide (DMF, Sigma-Aldrich, ≥99.8%), and dichloromethane (DCM, Sigma-Aldrich, ≥99.5%) were purified by Pure Process Technology (Solvent Purification System, SPS), and other solvents were purchased and used without further purification: chloroform (CHCl_3_, Sigma-Aldrich, ≥99%), 1,4-dioxane (Sigma-Aldrich, 99.8%), diethyl ether (Et_2_O, Fisher Scientific, ≥99%), dimethylacetamide (DMAc, Sigma-Aldrich, ≥99%), dimethyl sulfoxide (DMSO, Fisher Scientific, ≥99.9%), hexane (Fisher Scientific, ≥ 98.5%), and heptane (Fisher Scientific, ≥96%). Dimethyl sulfoxide-*d*_6_ (D, 99.9%), deuterium oxide (D, 99.9%), and chloroform (D, 99.8%) were purchased from Cambridge Isotope Laboratories. Glycopolymers were prepared and characterized as previously described.^33,35^ CTB was purchased from Sigma-Aldrich (C9903, United States). To avoid the risks of toxic infection, in this paper, we used only CTB to mimic the actual binding interaction. The control molecules for SPR, GM_1_ pentasaccharide sodium salt, and Lewis Y trisaccharide were purchased from Enzo Life Sciences and Sigma-Aldrich, respectively. SPR materials, including a Biacore CM5 sensor chip, an amide coupling kit, 10 mM Na-acetate immobilization buffer, and P20 surfactant, were purchased from Cytiva. Running buffer (10 mM HEPES pH 7.4, 150 mM NaCl, 3 mM EDTA, and 0.05% v/v P20) was freshly made and filtered through sterile 0.2 μm filters before use.

### Experimental Methods

Norbornenyl glycopolymers were produced following previously published methods (see Supporting Information).^33,35^ NMR spectra of the glycopolymer and monomers were acquired on Bruker Bruker Avance III 700 (^1^H: 700 MHz; ^13^C: 176 MHz), 500 (^1^H: 500 MHz; ^13^C: 125 MHz), and Bruker Nanobay 400 (^1^H: 400 MHz; ^13^C: 100 MHz) spectrometers. Chemical shifts are reported in parts per million (ppm) relative to residual solvent signals.

#### Gel Permeation Chromatography (GPC)

Gel permeation chromatography (GPC) measurements were carried out by using a Shimadzu system consisting of an SCL-10A system controller, an LC-20AT pump, and a CTO-10AS column oven. Separation was achieved with two Phenogel columns connected in series (5 μm, 50 Å, 300 × 4.6 mm, 100–3k; and 5 μm, 10^3^ Å, 300 × 4.6 mm, 1k–75k), and detection was performed using a Brookhaven Instruments BI-DNDC differential refractive index detector. Filtered HPLC-grade tetrahydrofuran (THF) was used as the eluent. Protected polymer samples were prepared in THF and passed through a 0.45 μm PTFE filter prior to injection (100 μL). Chromatographic analysis was conducted at 30 °C with a flow rate of 0.35 mL min^–1^. Molecular weight distributions were determined by using polystyrene (PS) standards.

#### Surface Plasmon Resonance (SPR)

SPR affinity and kinetic measurements were conducted using a Biacore T200 (Cytiva) with a CM5 sensor chip. Each experiment utilized 2 flow channels, including a CTB-immobilized channel and a blank reference channel for background subtraction. CTB (30 μg/mL) was dissolved in sodium acetate buffer (10 mM, pH 4.5) and immobilized on a Biacore Series S Sensor Chip CM5 (BR100530, Cytiva, Uppsala, Sweden) using amine coupling reagents (1-ethyl-3-(3-(dimethylamino)­propyl)­carbodiimide hydrochloride (EDC), *N*-hydroxysuccinimide (NHS), and 1.0 M ethanolamine-HCl pH 8.5, BR100633) at varying surface densities (1000–3000 RU), enabling evaluation of binding behavior across different ligand densities. The binding kinetics of each polymer were tested at 25 °C by flowing three to five different concentrations (ranging from 10 nM (0.3–0.7 μg/mL) to 7 μM (200–490 μg/mL)) in SPR running buffer (10 mM HEPES pH 7.4, 150 mM NaCl, 3 mM EDTA, and 0.05% v/v P20) at a flow rate of 30 μL/min. An association phase of 90–180 s was used, followed by a dissociation phase of 180 s, and the chip surface was regenerated by injecting 10 mM NaOH for 60 s. Buffer injections served as blank controls and were used in double referencing, which was performed by subtracting both the reference channel signal and buffer-only injections.^36^ All binding data were analyzed using Biacore T200 Evaluation software 3.2 (Cytiva) and fitted to a heterogeneous ligand kinetic model. The use of the 1:1 binding model failed to adequately fit the CTB–glycopolymer interactions. Additional SPR kinetic experiments were performed for all polymers, adjusting their concentrations based on molecular weight stoichiometry to achieve equivalent SPR responses based on the initial kinetic results.

The stability of the polymer–CTB complexes is inferred from the dissociation rate constant *k*_d_. A low *k*_d_ value indicates high stability of the complex and is used to calculate the residence time of the interaction (τ) and the half-life (*t*_1/2_).^37–39^

1 $$t_{1/2}^{\mathrm{dissoc}.}=\frac{0.693}{k_{\mathrm{off}}}$$

## Results and Discussion

We produced norbornenyl glycopolymers using ruthenium-catalyzed ring-opening metathesis polymerization (Scheme 1; see Supporting Information for detailed methods and spectra). Due to ruthenium’s outstanding functional group tolerance and high reproducibility,^47^ we were able to obtain well-controlled molecular weights and narrow dispersities. Glycopolymer analytes were synthesized and characterized (Table 1; Figures S1–S21). Three different homopolymers: pGal_100_, pFuc_100_, and pGlc_100_, an equimolar mixture of pGal_100_ and pFuc_100_, and random copolymers: pGal_15_Fuc_15_, pGal_50_Fuc_50_, pGal_25_Fuc_75_, pGal_75_Fuc_25_, pGal_15_Fuc_15_Glc_70_, pGlc_50_Fuc_50_, and pGlc_50_Gal_50_ were prepared. These polymers were analyzed in their protected forms using NMR and GPC (Table 1; Figure 2). After deprotection, they were stored as lyophilized powders and reconstituted right before SPR testing.

**Figure 2:**
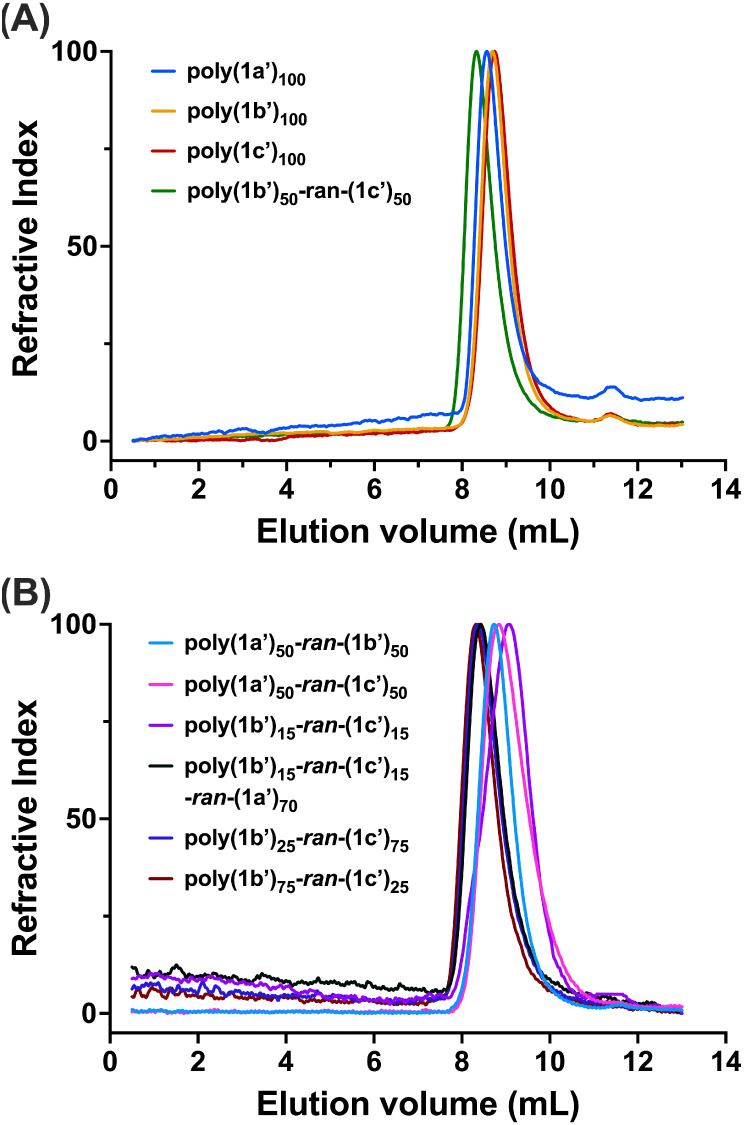
Gel permeation chromatography (GPC) chromatograms for protected norbornenyl glycopolymers in THF. (A) poly­(**1a′**)_100_, poly­(**1b′**)_100_, poly­(**1c′**)_100_, poly­(**1b′**)_50_-*ran*-(**1c′**)_50_. (B) poly­(**1a′**)_50_-*ran*-poly­(**1b′**)_50_, poly­(**1a′**)_50_-*ran*-poly­(**1c′**)_50_, poly­(**1b′**)_15_-*ran*-poly­(**1c′**)_15_, poly­(**1b′**)_15_-*ran*-poly­(**1c′**)_15_-*ran*-poly­(**1a′**)_70_, poly­(**1b′**)_25_-*ran*-poly­(**1c′**)_75_, poly­(**1b′**)_75_-*ran*-poly­(**1c′**)_25_.

**Table 1:** Molecular Weight Data for Protected Norbornenyl Glycopolymers

Polymer	*M*_n_, theor	*M*_n_, GPC^a^	*M*_w_, GPC^a^	Đ^b^	DP^c^
poly(**1a′**)_100_	51,300	47,310	53,230	1.12	90
poly(**1b′**)_100_	51,300	41,630	46,830	1.13	133
poly(**1c′**)_100_	45,500	39,610	44,440	1.12	141
poly(**1b′**)_15_-*ran*-poly(**1c′**)_15_	14,655	29,540	32,800	1.11	54
poly(**1b′**)_50_-*ran*-poly(**1c′**)_50_	48,400	64,360	73,310	1.14	173
poly(**1a′**)_50_-*ran*-poly(**1b′**)_50_	51,300	43,450	47,890	1.10	163
poly(**1a′**)_50_-*ran*-poly(**1c′**)_50_	48,400	36,350	40,940	1.13	143
poly(**1b′**)_15_-*ran*-poly(**1c′**)_15_-*ran*-poly(**1a′**)_70_	50,430	54,050	58,730	1.09	120
poly(**1b′**)_25_-*ran*-poly(**1c′**)_75_	46,950	64,070	72,580	1.13	143
poly(**1b′**)_75_-*ran*-poly(**1c′**)_25_	49,850	70,080	78,930	1.13	112

a Molecular weights were calculated using GPC and a refractive index detector calibrated with polystyrene (PS) in tetrahydrofuran (THF) with a flow rate of 0.35 mL/min. *M*_n_ is the number-average molecular weight; *M*_w_ indicates the weight-average molecular weight.

b Đ is defined as the dispersity of the glycopolymers measured by GPC.

c DP, the number of repeating units of the glycopolymer, is determined from the proton NMR integration ratio of the phenyl end group to the anomeric proton.

**Scheme 1:**
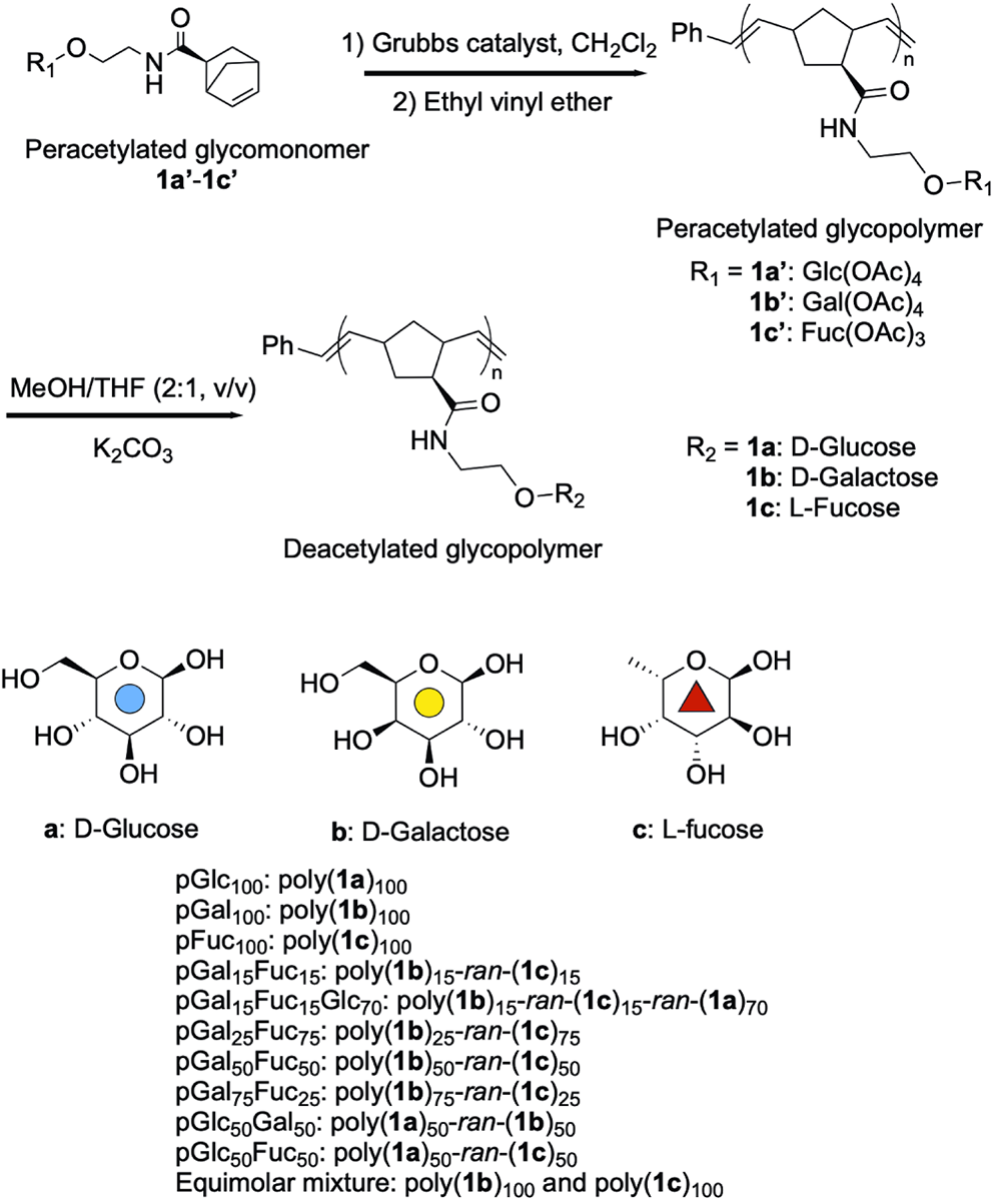
Norbornenyl Glycopolymers Synthesized via Ring-Opening Metathesis Polymerization (ROMP)^33,35^

Glycomonomers were designed to target both the canonical and noncanonical binding sites, with β-d-galactose and α-l-fucose serving as the representative ligands. Each binding site presents different affinities (58 nM for GM_1_ and 1.4 mM for LeY tetrasaccharide).^12^ β-d-glucose was included as a negative control that does not bind to any of the binding sites (Figure 3D).^34^

**Figure 3:**
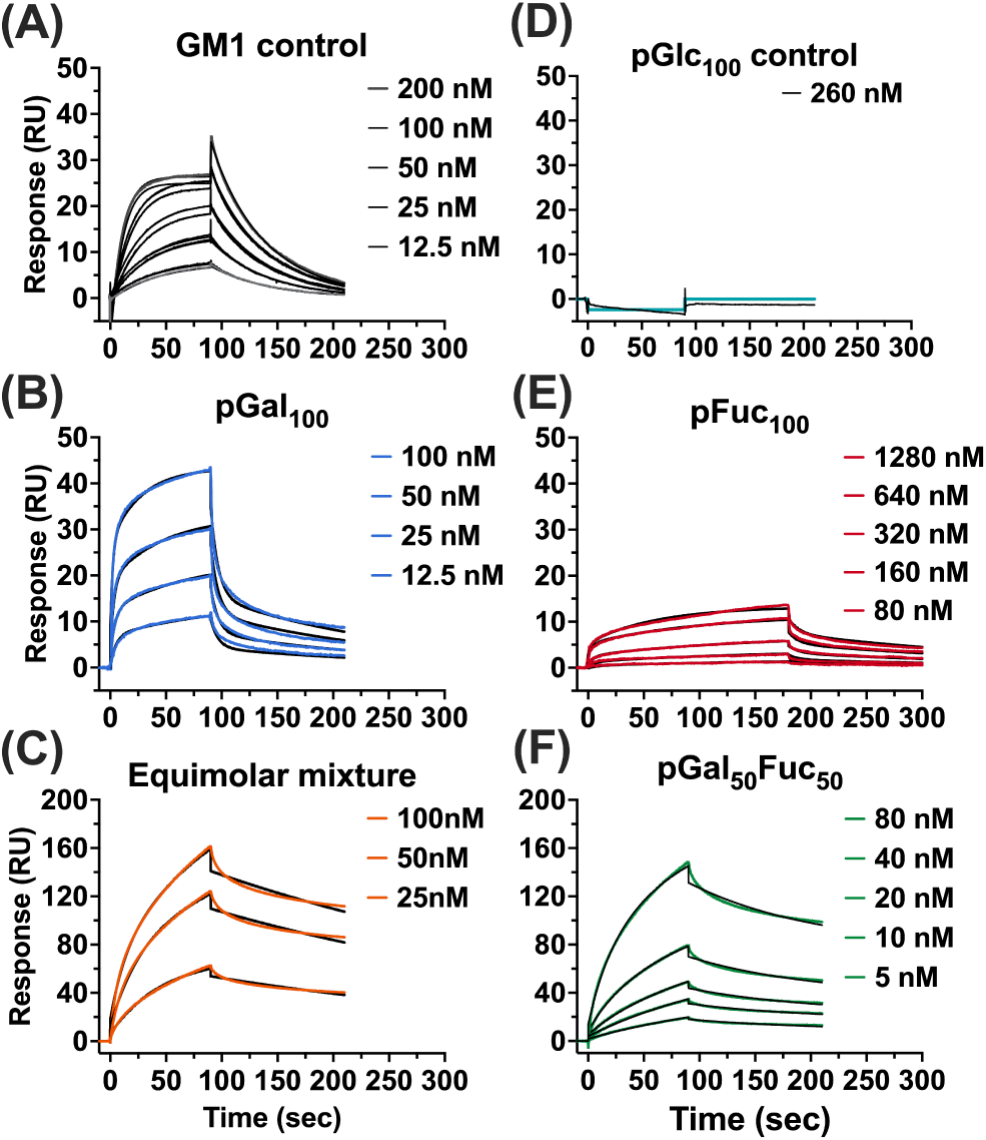
Representative sensorgrams for norbornenyl glycopolymer–CTB binding kinetics. Sensorgrams were measured by SPR at different concentrations of the glycopolymer. Data were fit to the heterogeneous ligand model (black lines). All analytes were tested at least in duplicate.

The binding interaction kinetics were analyzed using the Biacore T200 SPR system, with an experimental setup designed to first immobilize CTB (Scheme 2). Our target protein, CTB, was immobilized on a carboxymethylated dextran surface (CM5 chip) using EDC-NHS-mediated amine coupling at pH 4.5 and at varying surface densities (1000–3000 RU), enabling evaluation of binding behavior across different ligand densities. GM_1_ pentasaccharide sodium salt served as a positive binding control to validate the functionality of the CTB-coated surface. The results displayed identical and consistent binding kinetics and affinity compared to previous reports.^12,40,41^ Glycopolymers were flowed over the surface at various concentrations to account for the differing affinities of the carbohydrate ligands.

**Scheme 2:**
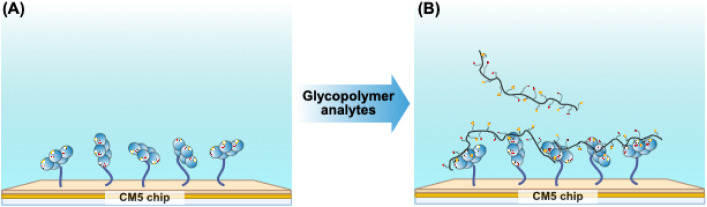
Biacore T200 SPR System Used to Test Glycopolymer–CTB Binding Kinetics. (A) CTB Was Covalently Attached to CM5 Chips and (B) Each Type of Glycopolymer Was Allowed to Migrate across the Derivatized Chip and Binding and Release Were Measured as a Function of Time

### Investigating the Binding Activity of CTB with Norbornenyl Glycopolymers

In the sensorgrams, the association phase (left-hand rising curve) reflects the binding of glycopolymers to immobilized CTB during injection. The dissociation phase (right-hand falling curve) starts when the analyte is replaced by a buffer, revealing how quickly the complexes dissociate. The sensorgram of each glycopolymer was measured and performed at a minimum in duplicate. Including the positive control GM_1_ pentasaccharide sodium salt, all of the glycopolymers displayed a clear concentration-dependent association with CTB, increasing in response units during the association phase. Upon introduction of running buffer, a gradual decrease in RU for surface regeneration was observed, consistent with the dissociation of the CTB–glycopolymer complex. The CTB-immobilized surface was successfully regenerated between cycles, returning to the baseline RU, confirming the reversible binding of CTB to glycopolymers.

We noticed that in the association phase, pGal_50_Fuc_50_ and equimolar mixture binding to CTB did not reach saturation, and both glycopolymer–CTB complexes reached ∼160 RU, much higher than that for other glycopolymers, indicating that a high mass of glycopolymer was accumulated on the CTB-immobilized surface. pGlc_100_, the negative control that does not bind to CTB, did not reflect any RU after injection, as expected.

In the dissociation phase, pGal_50_Fuc_50_ and the equimolar mixture showed the same dissociation trend, and treated chips were hard to regenerate compared to other polymers. Each sensorgram indicates the unique binding mechanism that can be derived from the multivalent structure of glycopolymers and the presentation of two distinct sugar ligands. The association and dissociation rate constants were obtained by global or local fitting of the sensorgrams to the heterogeneous ligand model. We fit the interaction to a model that includes multiple steps of interactions because the multivalency of glycopolymers leads to multiple binding events with CTB.

Surface density can influence mass transport effects, and to that effect, we chose to use a higher flow rate of 30 μL/min to minimize the likelihood of mass transport limitation. All included sensorgrams have *tc* values (flow rate-independent component of the mass transfer constant) either equal to or exceeding the recommended 10^8^.^42,43^ While transport can influence association rates, it does not account for the prolonged dissociation observed, which is attributed to multivalent binding.

### Investigating the Kinetic Binding between CTB and Different Glycopolymers

The kinetics of glycopolymer (GM_1_, pGlc_100_, pGal_100_, pFuc_100_, equimolar mixture of pGal_100_ and pFuc_100_, and random copolymer pGal_50_Fuc_50_) binding were measured in the low to mid-nanomolar concentration range (6.25–520 nM, Figure 3). To uncover the detailed kinetic mechanisms, we evaluated their affinity and on/off rates to elucidate the binding mechanism and to estimate the effective binding duration as an assessment of glycopolymer–CTB complex stability under dynamic conditions.

To ensure binding occurs, we utilized a high CTB density surface. The monovalent ligand GM_1_ demonstrated identical binding affinity compared to previous studies,^33^ which confirms that the CTB was intact on the surface. The binding was consistent with a 1:1 binding model, and GM_1_ exhibited a dissociation constant (*K*_D_) of 55.0 ± 1.2 nM, determined from a *k*_on_ of 4.27 × 10^5^ ± 8.91 × 10^3^ M^–1^ s^–1^ and a *k*_off_ of 2.35 × 10^^–2^^ s^–1^.

For polymeric ligands, binding kinetics can deviate from monovalent behavior due to the presence of multiple sugar units along the polymer backbone. To account for these effects, the kinetic data were analyzed using the heterogeneous ligand model for lectin–glycopolymer interactions (Figure 4). According to the model, two independent series of binding steps (*k*_1_ and *k*_1_′) occurred in parallel, indicating the presence of two or more types of binding interactions.

**Figure 4:**
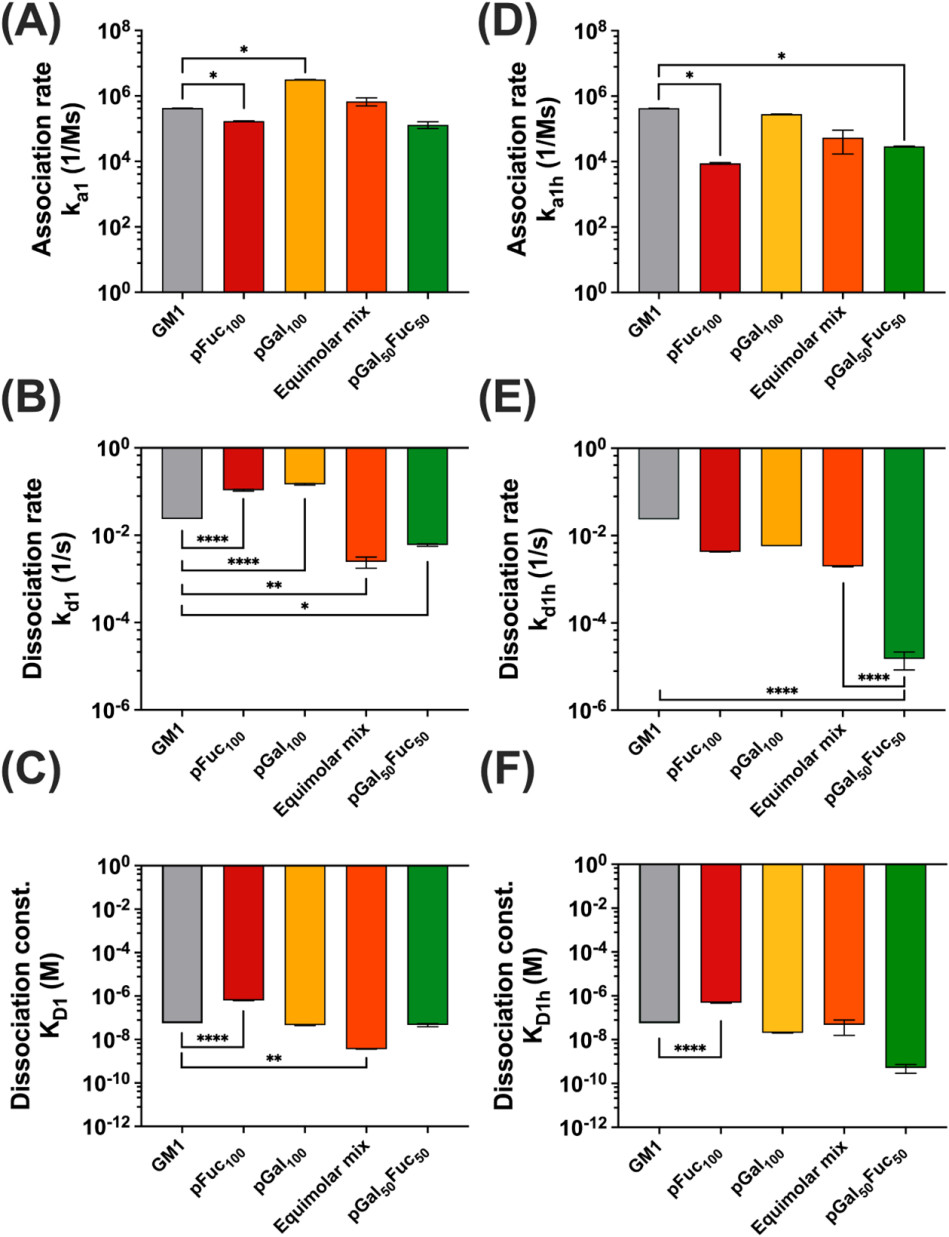
Kinetic parameters for glycopolymers with CTB were obtained from SPR. (A–C) Monovalent-binding association rate *k*_a1_, dissociation rate *k*_d1_, and equilibrium dissociation constant *K*_D1_. (D–F) Multivalent-binding association rate *k*_a1h_, dissociation rate *k*_d1h_, and equilibrium dissociation constant *K*_D1h_. Statistical comparison between GM_1_ and each polymer’s *k*_on_/*k*_off_ rate, and dissociation constant was performed using one-way ANOVA. Asterisks denote statistical significance: no significance not shown, *p < 0.05, **p < 0.01, ***p < 0.001, ****p < 0.0001 for all comparisons. Data are presented as mean ± standard deviation (SD) (*n* = 2).

We observed distinct differences in kinetics and affinity between the two inferred binding modes. The data suggest fast-associating and fast-dissociating components, likely corresponding to a monovalent interaction similar to the GM_1_-binding mechanism (*k*_1_, Figure 4A–C). In addition, a slower binding and dissociation phase occurs, which is assumed to represent multivalent interactions (*k*_1h_, Figure 4D–F) involving multiple simultaneous contacts. The monovalent interaction exhibits faster binding but lower binding affinity, while the multivalent component contributes to binding stability and stronger overall binding.

Homopolymers exhibited fast association and dissociation rates in the monovalent binding mode. For example, pGal_100_ has a 7.5-fold faster binding rate (*k*_a1h_) than GM_1_, but a 5-fold faster release rate (*k*_d1h_) compared to that of the GM_1_ pentasaccharide, indicating reduced complex stability. A similar trend was observed for pFuc_100_, which bound more slowly than GM_1_ and dissociated more rapidly. Fucose is known to have lower intrinsic affinity for CTB than galactose,^41^ and the polymer SPR results matched our forecast, showing lower binding affinity.

In contrast, the equimolar mixture and the random copolymer pGal_50_Fuc_50_—both presenting galactose and fucose simultaneously—did not show substantial improvement in association rate or overall affinity compared to GM_1_ but exhibited significantly slower dissociation rates (**, *p* < 0.01 for equimolar mixture; *, *p* < 0.05 for pGal_50_Fuc_50_; Figure 4B). These results indicate that at comparable affinity levels (*K*_D1_, Figure 4C), homopolymer–CTB complexes are less stable in the monovalent binding mode, whereas incorporating both galactose and fucose (either in an equimolar mixture or in a single copolymer) enhances stability.

In the multivalent binding phase, all polymers displayed reduced association rates relative to GM_1_, reflecting slower interaction kinetics than in the monovalent mode, with no significant differences in affinity (Figure 4F). Notably, the random copolymer pGal_50_Fuc_50_ showed a dramatically reduced dissociation rate (*k*_d1_, ****, *p* < 0.0001), indicating a strong increase in stability. We attribute this stability to the heteroligand structure, which is consistent with our previous study, in which large aggregates were observed for pGal_50_Fuc_50_ by both DLS and TEM imaging.^34^ The results clearly delineate that the utilization of both ligands on the same polymer structure is crucial for a strong multivalent interaction. Consistent with the requirement for a copolymer structure, this increase in stability in the multivalent phase did not occur with the equimolar mixture, which presents the two essential sugar ligands on different polymer chains. To further investigate factors influencing pGal_50_Fuc_50_ stability and complex formation, we examined the surface density, sugar valency, and tested additional glycopolymer controls.

### Random Copolymer–CTB Stability Depends on the Density of CTB Immobilized on the Surface

In a conventional 1:1 stoichiometric binding interaction, binding kinetics are typically independent of the immobilized ligand surface density. For multivalent interactions, both mono- and multivalent binding activity can be significantly affected by surface CTB density due to statistical rebinding events, as well as multivalent points of contact.

As shown in Figure 5, we observed that while both the random copolymer pGal_50_Fuc_50_ and the equimolar polymer mixture (pGal_100_ + pFuc_100_) reached comparable relative RU values, the equimolar polymer mixture exhibited lower stability due to its fast dissociation phase, as reflected by a high *k*_d1_. To further investigate this phenomenon, we analyzed binding kinetics with different CTB surface densities.

**Figure 5:**
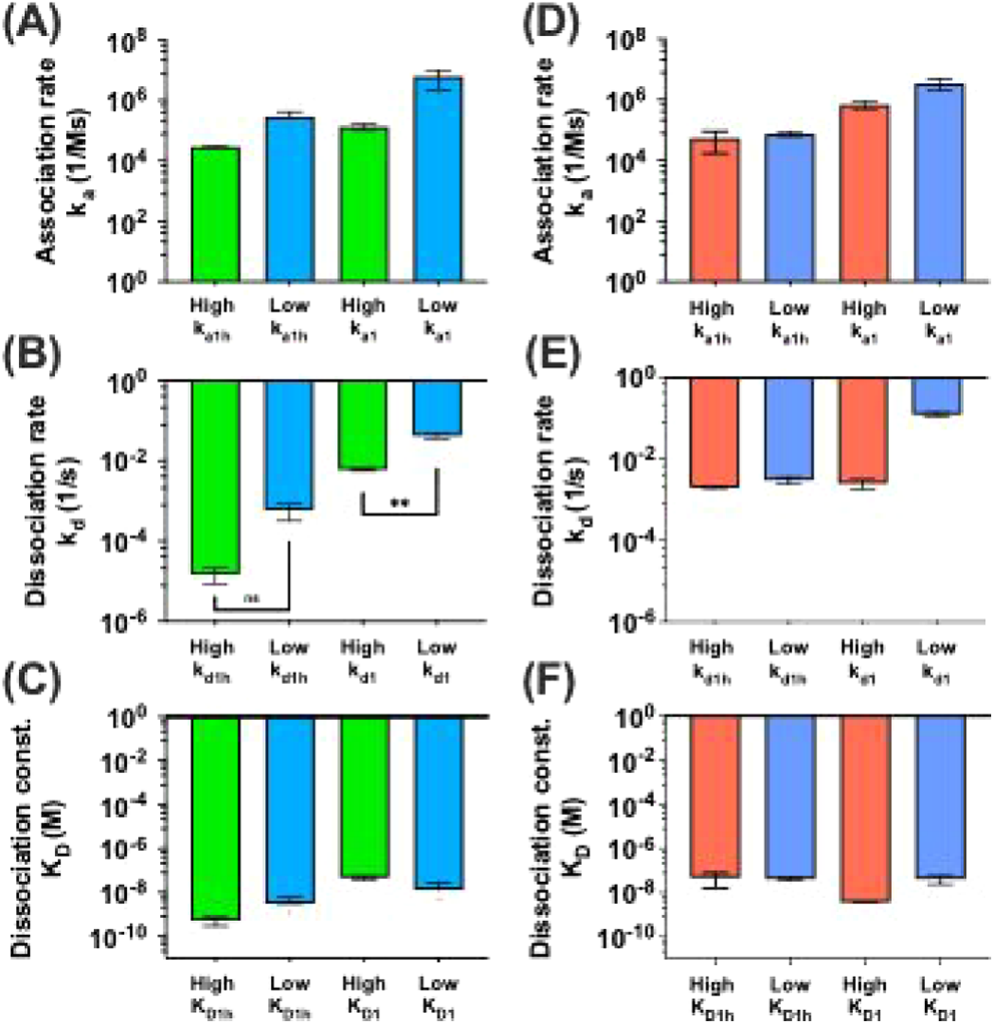
Polymer–CTB binding constants. pGal_50_Fuc_50_ (A–C) and equimolar mixture (D–F) kinetic constants for binding to low and highCTB density surfaces. Polymers were applied to the low (1000 RU) and high (3000 RU) density surfaces with the same flow rate and time shift. Data are presented as mean ± standard deviation (SD) (*n* = 2).

Across both low (1000 RU) and high (3000 RU) CTB density surfaces, the association rate for the monovalent component (*k*_a1_) was consistently faster than that of the multivalent component (*k*_a1h_). The corresponding dissociation rates (*k*_d1_) indicated that monovalent interactions were short-lived, although dissociation from the high-density surface was an order of magnitude slower compared to dissociation from the low-density surface. In contrast, for pGal_50_Fuc_50_ the multivalent component showed a significantly (2 orders of magnitude) slower dissociation rate (*k*_d1h_) from the high-density surface relative to the low-density surface, indicating that stable complex formation depends on the density of CTB units. This same stability increase was not observed with the equimolar mixture. Moreover, the stability of the pGal_50_Fuc_50_ on the low-density CTB surface was still higher than that of the equimolar mixture on either a high- or low-density CTB surface. This stability suggests that the random copolymer engages CTB robustly regardless of the surface ligand abundance.

In contrast, the equimolar polymer mixture dissociation rate displayed a clear dependence on surface density; however, there was no dependence of the multivalent phase kinetic stability on CTB density. This suggests that while the polymer mixture may access multivalent binding modes more effectively at higher ligand densities, the resulting complexes are less stable, potentially due to suboptimal spatial alignment or lower cooperative binding strength. Overall, the random copolymer demonstrated more stable and efficient binding to CTB at varying surface densities, even at a low CTB density.

The density-dependent behavior indicated that a prolonged residence time is provided by the multivalent effect of pGal_50_Fuc_50_. The equimolar polymer mixture failed to form stable complexes even under favorable conditions. These findings highlight the advantage of simultaneous sugar display within a single polymer chain, making random copolymers a more effective design for achieving robust CTB binding and complex stabilization. To better reflect physiological conditions and evaluate the performance limits of our glycopolymers, we therefore conducted the remaining assays using low-density CTB surfaces.

### CTB–Glycopolymer Composition–Function Relationships Required to Form a Stable Complex

After establishing the influence of polymer structure and surface density on CTB binding, we next asked if the spatial orientation of sugar ligands and the ratio of galactose and fucose on the same polymer chain affect the kinetic process. First, we synthesized control copolymers bearing either galactose or fucose as a random mixture with glucose, which does not bind to CTB, pGlc_50_Gal_50_, and pGlc_50_Fuc_50_, as single binding-sugar controls to test whether pGal_50_Fuc_50_ stability derives from reduced density of binding ligand, or from having two binding ligands in a single polymer chain (see Supporting Information).

The binding of pGlc_50_Fuc_50_ and pGlc_50_Gal_50_ to CTB did not show a large difference between the monovalent and multivalent modes and also failed to fit a 1:1 binding model, indicating that the multivalent and statistical effects observed with the random pGal_50_Fuc_50_ copolymer are diminished for the single binding-sugar copolymers (Figure 6). These results strongly imply that galactose and fucose are both responsible for stable complex formation. Testing these polymers allowed us to isolate the effect of copresenting galactose and fucose within a single scaffold.

**Figure 6:**
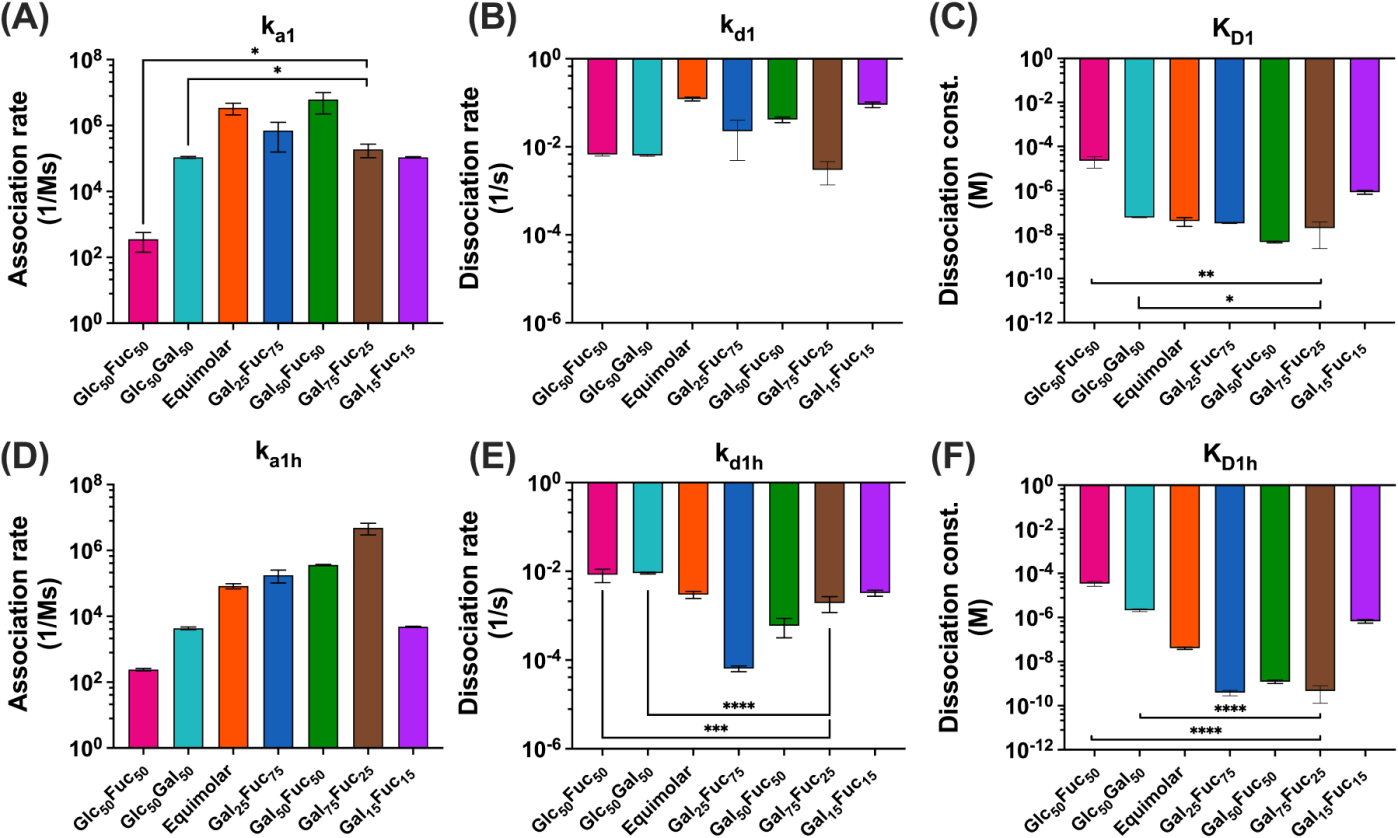
Composition–function relationships for sugar ligands presented on the same polymer backbone evaluated on a low-density CTB surface. Panels (A–C) present monovalent rate constants (*k*_1_), while panels (D–F) present multivalent rate constants (*k*_1h_). A low CTB density (1000 RU) chip was used throughout this study. Each sample was measured in duplicate. Statistical comparison between single binding-sugar controls: pGlc_50_Fuc_50_ and pGlc_50_Gal_50_ to *k*_on_/*k*_off_ rate, and dissociation constant of each glycopolymer was performed using one-way ANOVA. Asterisks denote statistical significance: no significance not shown, *p < 0.05, **p < 0.01, ***p < 0.001, ****p < 0.0001 for all comparisons. Data are presented as mean ± standard deviation (SD) (*n* = 2).

Next, we evaluated how chain length (DP) and per-chain ligand density influence random copolymer binding by comparing pGal_50_Fuc_50_ to two lower-valency analogs and to the single binding-sugar controls pGlc_50_Fuc_50_ and pGlc_50_Gal_50_ (Figure 6). The first analog, pGal_15_Fuc_15_Glc_70_, has the same DP as the 100-mer class but a much lower fraction of binding ligands; the second, pGal_15_Fuc_15_, has the same total valency but is shorter and has a higher local density because no glucose spacers are present (Figure 6).

pGal_15_Fuc_15_Glc_70_ showed very low RU, and sensorgrams could not be fit to the heterogeneous-ligand model (no kinetic parameters and sensorgram, see Figure S22), consistent with falling below the local-density threshold for multivalent capture. In contrast, pGal_15_Fuc_15_ displayed moderate association rates in both phases (*k*_a1_ and *k*_a1h_) that were not significantly different from either single binding-sugar control (nanogroups not shown). The pGal_15_Fuc_15_ monovalent dissociation rate (*k*_d1_) was significantly faster than the rates for both controls (****, *p* < 0.0001, Figure 6B), indicating reduced short-lived stability. Interestingly, its multivalent dissociation had a small but statistically significant decrease (*k*_d1h_, ****, *p* < 0.0001, Figure 6E), suggesting that increased local density (shorter chain without glucose spacers) confers a modest avidity benefit, although not enough to offset the instability seen in the monovalent phase.

These results indicate first that polymer sugar density dictates the binding capacity. When the binding ligand spacing averages 2–3 monomer units (pGal_15_Fuc_15_Glc_70_), no binding was observed. With increased density and shorter overall length (pGal_15_Fuc_15_), no significant change in association rates in either phase (*k*_a1_ or *k*_a1h_) was observed, but the monovalent dissociation rate was faster than the single binding-sugar control (****, *p* < 0.0001, Figure 6B), indicating reduced, i.e., short-lived, stability. In the multivalent phase, the change in dissociation was small (*k*_d1h_, Figure 5E), relative to pGal_50_Fuc_50_ at low CTB density, and did not reach significance (see Figure 6E–F), consistent with a modest avidity benefit that does not fully offset the instability of the monovalent component. This kinetic pattern aligns with our prior aggregation study, which showed only small or minimal aggregates for pGal_15_Fuc_15_.^33^

As low valency and larger spacing produced suboptimal kinetics, we focused next on long, high-valency (100-mer) random copolymers and tuned the Gal:Fuc ratio (25:75, 50:50, 75:25) building on the pGal_50_Fuc_50_ benchmark (kinetic data shown in Figure 6; for sensorgram, see Figure S22). We asked whether composition alone could modulate the rate or the stability trade-off. In the multivalent phase, increasing the fucose fraction (pGal_25_Fuc_75_) led to slower *k*_a1h_ and *k*_d1h_ (Figure 6E) with slight or no effect on the overall stability. Conversely, increasing the galactose fraction gave faster *k*_a1h_ and *k*_d1h_, implying rapid rebinding and maintenance of stability (Figure 6E). Notably, pGal_75_Fuc_25_ showed a marked RU spike immediately after injection (see Supporting Information), consistent with rapid rebinding by galactose, yet this kinetic acceleration did not translate into an improved *K*_D_ (Figure 6C–F). In the monovalent phase, changes in *k*_d1_ for the ratio-extreme copolymers (25:75 and 75:25) were not significant relative to those of the controls.

Together, these findings support that within sufficiently long, high-valency chains, the fucose ligand enriches stability (reduced *k*_d1_, but not *k*_d1h_), whereas the galactose ligand accelerates rebinding, yielding similar affinity in both binding modes when both ligands are codisplayed. These findings support the view that composition tunes kinetics while leaving affinity largely unchanged across Gal/Fuc random copolymers.

### Relevance of Kinetic Parameters to Physiologic Conditions

Once we established the relationship between CTB–glycopolymer aggregation and inhibitory efficacy, a deeper understanding of CTB–glycopolymer binding and aggregation kinetics with different ligands and polymer architectures is essential for translating these findings to physiological applications. It is important to note that our kinetic data were obtained using a surface plasmon resonance system, in which CTB was covalently immobilized on a carboxymethylated dextran surface with fixed conformation and multiple orientations, where we activated and labeled the lysine functional groups for covalent bond formation (Figure S23). This configuration facilitates kinetic analysis between CTB and glycopolymers, but it does not fully recapitulate the natural intestinal conditions, where CTB is secreted as cholera toxin (CTX) and interacts with glycans on the epithelial cell surface under dynamic fluid flow conditions.^44,45^

Although the SPR setup provides valuable mechanistic insight into ligand–receptor interactions and enables direct comparisons between carbohydrate compositions, our design lacks physiological parameters to mimic human gut conditions. Therefore, complementary *in vitro* and *in vivo* models—such as cellular monolayers,^46–49^ intestinal mucus,^50,51^ intestinal organoids,^52,53^ or *ex vivo* gut tissue^54^—will be required to further validate these mechanistic insights and establish translational relevance.

In addition, we calculated the polymer concentrations based on theoretical molecular weight with confirmed narrow dispersity to minimize the noise from batch-to-batch variations and allow for consistent comparisons across polymer series with different architectures and ligand compositions. While actual molecular weights may vary slightly, this standardized calculation ensures that observed binding trends reflect structural and compositional differences rather than mass-related effects. We acknowledge that using theoretical molecular weights may introduce minor deviations from actual chain lengths; however, this method was applied uniformly across all polymer samples to ensure that binding trends primarily reflect differences in polymer structure and ligand presentation.

For our ultimate goal of contributing to a clinical solution, pharmacokinetics (PK) and pharmacodynamics (PD) are essential in determining a drug’s clinical performance and efficacy, with evaluations conducted in either open or closed systems.^38,55^ Open-system measurements like SPR better approximate *in vivo* conditions, where drug concentrations fluctuate due to dilution, metabolism, and elimination. For orally administered glycopolymers, elimination is a key determinant, as these large macromolecules are unlikely to be absorbed and must act locally within the time-limited window of intestinal transit. In healthy adults, this window ranges from 3 to 8 h, but in diarrheal conditions, it may be shortened to under 3 h.^56,57^

Historically, efforts to enhance drug–target interaction have focused on minimizing the dissociation rate constant (*k*_off_), thereby prolonging residence time and increasing target occupancy.^38^ In our study, pGal_50_Fuc_50_ showed high stability only at high CTB density. pGal_25_Fuc_75_ achieved a dissociation half-life reaching 2.5–3 h under low-density conditions, fitting closely to the gastrointestinal transit time (Table 2). The kinetic durability supports its potential as a viable oral therapeutic for toxin sequestration.

**Table 2:** Half-Life Prediction Is Based on SPR Equilibrium Dissociation Constants (*K*_D1_ and *K*_D1h_)­^a^,^b^

High CTB density half-life				
	Monovalent binding (*k*_1_)		Multivalent binding (*k*_1h_)	
Analyte (abbreviation)	*t*1/2 (min)	*K*_D1_ (nM)	*t*1/2 (min)	*K*_D1h_ (nM)
GM_1_	0.49	55.9	–	–
poly(**1b**)_100_	<10	45.9 ± 2.3	<10	20.2 ± 0.2
poly(**1c**)_100_	<10	629 ± 17	<10	482 ± 28
Equimolar poly(**1b**)_100_+poly(**1c**)_100_	<10	3.59 ± 0.03	<10	47.5 ± 31.7
poly(**1b**)_50_-*ran-* poly(**1c**)_50_	<10	46.3 ± 7.8	854 ± 377	0.51 ± 0.22

Low CTB density half-life				
	Monovalent binding (*k*_1_)		Multivalent binding (*k*_1h_)	
Analyte (abbreviation)	*t*1/2 (min)	*K*_D1_ (nM)	*t*1/2 (min)	*K*_D1h_ (nM)
poly(**1a**)_50_-*ran-* poly(**1c**)_50_	<10	22500 ± 1200	<10	34400 ± 8600
poly(**1a**)_50_-*ran-* poly(**1b**)_50_	<10	60 ± 1	<10	2130 ± 300
poly(**1b**)_15_-*ran-* poly(**1c**)_15_	<10	840 ± 170	<10	675 ± 120
poly(**1b**)_25_-*ran-* poly(**1c**)_75_	<10	32 ± 1	182 ± 27	0.39 ± 0.11
poly(**1b**)_50_-*ran-* poly(**1c**)_50_	18 ± 1	4.6 ± 0.4	27 ± 6	1.2 ± 0.2
poly(**1b**)_75_-*ran-* poly(**1c**)_25_	<10	20 ± 17	<10	0.46 ± 0.34

a Half-life (*t*_1/2_) represents the time required for 50% of the bound analyte to dissociate from the ligand and is a reflection of the stability of the complex.

b Equilibrium dissociation constant (*K*_D_) is defined as the ratio of the dissociation rate constant (*k*_–1_) to the association rate constant (*k*_1_).

Mechanistically, this prolonged residence time is linked to the cooperative, multivalent binding enabled by the simultaneous presentation of galactose and fucose on the same polymer chain. Homopolymers such as pGal_100_ or pFuc_100_—targeting only one of CTB’s glycan-binding sites—showed fast dissociation rates and unstable complex formation. Furthermore, control copolymers bearing glucose (pGlc_50_Gal_50_ and pGlc_50_Fuc_50_) demonstrated that the inclusion of a nonbinding sugar reduces the stability of the multivalent interaction, underscoring the necessity of functional ligand selection and heteroligand design. These findings emphasize the importance of spatial ligand presentation and heteromultivalency in optimizing CTB binding kinetics, consistent with recent reports demonstrating that AB_5_ toxins exhibit superselective, density-dependent binding behavior in a glycocalyx-mimetic system.^58^

Previously, we established the relationship between CTB–glycopolymer aggregation and inhibitory efficacy.^12,33,34^ In these studies, we demonstrated that CTB and glycopolymer form stable complexes only when glycopolymers simultaneously bind to two distinct sites that are on the same polymer scaffold, and the ratio between galactose and fucose is equally important. These findings highlight the importance of CTB–glycopolymer binding kinetics in optimizing drug–target interactions for blocking CT. The combination of high binding avidity, slow dissociation, and physiological stability achieved by pGal_25_Fuc_75_ makes it a strong candidate for future development as an oral inhibitor of cholera toxin.

## Conclusions

Our study presents a mechanistic and kinetic analysis of the binding of cholera toxin B subunit (CTB) to structurally defined norbornenyl-based glycopolymers synthesized via ROMP. Surface plasmon resonance revealed that polymer architecture, ligand density, and spatial arrangement critically influence CTB-binding stability.

Among the tested constructs, the random copolymer pGal_25_Fuc_75_ exhibited the slowest release time at low CTB density, with a markedly slow dissociation rate and prolonged half-life (Table 2). These data indicate cooperative multivalent interactions and the necessity to present both galactose and fucose, with fucose in higher abundance than galactose.

This kinetic advantage persisted under dynamic flow conditions simulating intestinal transit, further supporting pGal_25_Fuc_75_’s potential for therapeutic application. Our findings highlight the value of glycopolymer heterogeneity, spatial presentation, and kinetic tuning in designing effective CTB inhibitors, positioning pGal_25_Fuc_75_ as a promising lead for oral antitoxin therapies.

## Supplementary Material



## Notes

^1^H and ^13^C NMR spectra for compounds, additional experimental details, materials, and methods, additional SPR sensorgrams for polymers, and a figure of the CTB surface (PDF)

## Abbreviations

CTX: cholera toxin
CTA: cholera toxin A subunit
CTB: cholera toxin B subunit
ROMP: ring-opening metathesis polymerization
SPR: surface plasmon resonance
*k*_a1_: monovalent association rate
*k*_a1h_: multivalent association rate
*k*_d1_: monovalent dissociation rate
*k*_d1h_: multivalent dissociation rate
*K*_D1_: monovalent dissociation constant
*K*_D1h_: multivalent dissociation constant.
