# Supplementary material for: Kinetic Insights into Cholera Toxin B‑Biomimetic Glycan Interactions
Source: Biomacromolecules. 2026 Apr 7;27(5):3281–91. doi: 10.1021/acs.biomac.5c02759 (PMC13169385; doi:10.1021/acs.biomac.5c02759)
Supplement: Supplementary file 1 [file bm5c02759_si_001.pdf]

Supporting information for

Kinetic insights into cholera toxin B-biomimetic glycan interactions

*Kun Lin Hsieh,[1] Navanjalee T. Panagoda,[2] Jermaine L. Jenkins,[3] Nicole S. Sampson[1,*2]*

[1]Department of Chemistry, Stony Brook University, Stony Brook, New York 11794-3400, USA

[2]Department of Chemistry, University of Rochester, Rochester, New York 14627-0216, USA

[3]Department of Biochemistry and Biophysics, University of Rochester, Rochester, NY 14642-0001, USA

*To whom correspondence should be addressed: nicole.sampson@rochester.edu.

ORCIDs

Nicole S Sampson – 0000-0002-2835-7760;

Kun Lin Hsieh – 0000-0003-1427-9981

Navanjalee T. Panagoda − 0000-0003-2366-075X

Jermaine L. Jenkins – 0000-0003-2548-3275

Table of Contents

Glycopolymer synthesis: Ring-Opening Metathesis Polymerization (ROMP) S4
Poly(**1a'**)$_{50}$-*ran*-poly(**1b'**)$_{50}$ S5
Poly(**1a'**)$_{50}$-*ran*-poly(**1c'**)$_{50}$ S5
Poly(**1b'**)$_{15}$-*ran*-poly(**1c'**)$_{15}$ S5
Poly(**1b'**)$_{15}$-*ran*-poly(**1c'**)$_{15}$-*ran*-poly(**1a'**)$_{70}$ S6
Poly(**1b'**)$_{25}$-*ran*-poly(**1c'**)$_{75}$ S6
Poly(**1b'**)$_{50}$-*ran*-poly(**1c'**)$_{50}$ S6
Poly(**1b'**)$_{75}$-*ran*-poly(**1c'**)$_{25}$ S6
General deprotection method S7
Poly(**1b**)$_{15}$-*ran*-poly(**1c**)$_{15}$ S7
Poly(**1b**)$_{15}$-*ran*-poly(**1c**)$_{15}$-*ran*-poly(**1a**)$_{70}$ S7
Poly(**1b**)$_{25}$-*ran*-poly(**1c**)$_{75}$ S8
Poly(**1b**)$_{50}$-*ran*-poly(**1c**)$_{50}$ S8
Poly(**1b**)$_{75}$-*ran*-poly(**1c**)$_{25}$ S8
Figure S1. ^{1}H-NMR of poly(**1a'**)$_{50}$-*ran*-(**1b'**)$_{50}$ S9
Figure S2 ^{13}C-NMR of poly(**1a'**)$_{50}$-*ran*-(**1b'**)$_{50}$ S9
Figure S3. ^{1}H-NMR of poly(**1a'**)$_{50}$-*ran*-(**1c'**)$_{50}$ S10
Figure S4. ^{13}C-NMR of poly(**1a'**)$_{50}$-*ran*-(**1c'**)$_{50}$ S10
Figure S5. ^{1}H-NMR of poly(**1b'**)$_{15}$-*ran*-(**1c'**)$_{15}$ S11
Figure S6. ^{13}C-NMR of poly(**1b'**)$_{15}$-*ran*-(**1c'**)$_{15}$ S11
Figure S7. ^{1}H-NMR of poly(**1b'**)$_{15}$-*ran*-(**1c'**)$_{15}$-*ran*-(**1a'**)$_{70}$ S12
Figure S8. ^{13}C-NMR of poly(**1b'**)$_{15}$-*ran*-(**1c'**)$_{15}$-*ran-(***1a'**)$_{70}$ S12
Figure S9. ^{1}H-NMR of poly(**1b'**)$_{25}$-*ran*-(**1c'**)$_{75}$ S13
Figure S10. ^{13}C-NMR of poly(**1b'**)$_{25}$-*ran*-(**1c'**)$_{75}$ S13
Figure S11. ^{1}H-NMR of poly(**1b'**)$_{50}$-ran-(**1c'**)$_{50}$ S14
Figure S12. ^{13}C-NMR of poly(**1b'**)$_{50}$-ran-(**1c'**)$_{50}$ S14
Figure S13.^{1}H-NMR of poly(**1b'**)$_{75}$-*ran*-(**1c'**)$_{25}$ S15
Figure S14. ^{13}C-NMR of poly*(***1b'**)$_{75}$-*ran*-(**1c'**)$_{25}$ S15
Figure S15. ^{1}H-NMR of poly(**1a**)$_{50}$-*ran*-(**1b**)$_{50}$ S16
Figure S16. ^{1}H-NMR of poly(**1a**)$_{50}$-*ran*-(**1c**)$_{50}$ S17

Figure S17. ^{1}H-NMR of poly(**1b**)$_{15}$-*ran*-(**1c**)$_{15}$ S17
Figure S18. ^{1}H-NMR of poly(**1b**)$_{15}$-*ran*-(**1c**)$_{15}$-*ran*-(**1a**)$_{70}$ S18
Figure S19. ^{1}H-NMR of poly(**1b**)$_{25}$-*ran*-(**1c**)$_{75}$ S18
Figure S20. ^{1}H-NMR of poly(**1b**)$_{50}$-*ran*-(**1c**)$_{50}$ S19
Figure S21.^{1}H-NMR of poly(**1b**)$_{75}$-*ran*-(**1c**)$_{25}$ S19
Figure S22. SPR sensorgrams of copolymers. S20
Figure S23. Lysine distribution on the surface of CTB. S20

Glycopolymer synthesis: Ring-Opening Metathesis Polymerization (ROMP)

The previously published method for ROMP was followed:[1,2] glycomonomer (0.06 mmol, 30.7 mg) was dissolved in 0.3 mL CH_2Cl_2. To the reaction was added dichloro[1,3-bis(2,4,6-trimethylphenyl)-2-imidazolidinylidene](benzylidene)bis(3-bromopyri-dine)ruthenium(II) [3rd generation Grubbs catalyst] (6 µmol, 5.3 mg for the 10-mers and 0.6 µmol, 0.53 mg for the 100-mers) in CH_2Cl_2 (0.3 mL for the 10-mers and 0.7 mL for the 100-mers). The reaction was monitored by TLC until no monomer signal remained. Ethyl vinyl ether (0.1 mL) was added to quench the reaction when it was complete, and the mixture was stirred for an additional 30 min. The polymer was isolated by precipitation with cold Et_2O to yield 30- and 100-mers as brown to light brown sticky oils. The polymer was reprecipitated into cold diethyl ether (25 mL), and the suspension was centrifuged at 6,000 rpm for 5 min. The supernatant was decanted, and this procedure was repeated two more times. The polymer was allowed to dry overnight in vacuo.

Comparison of the integration values of the phenyl end cap moiety to the vinylic protons of the polymer backbone by ^1H NMR spectroscopy gave the degree of polymerization. Polydispersity indices (PDIs) and number-average molecular weights (M_n) were measured on a Brookhaven Instruments BI-DNDC refractometer, with a system comprised of Shimadzu SCL-10A controller, a Shimadzu LC-20AT pump, and a Shimadzu CTO-10AS column oven equipped with columns 5 µm 50 Å (300 × 4.6 mm, 100−3k) and 5 µm 10E3 Å (300 × 4.6 mm, 1k−75k) connected. The mobile phase used was HPLC-grade tetrahydrofuran (THF) filtered through a 0.2 µm nylon membrane and polystyrene polymer were used as standards.

NMR characterization of glycopolymers synthesized. Polymers poly(**1a**)$_{100}$, poly (**1b**)$_{100}$, and poly(**1c**)$_{100}$ were prepared as above and their spectra were consistent with the literature.[1] The NMR spectra of copolymers are listed below:

Poly(**1a'**)$_{50}$-*ran*-poly(**1b'**)$_{50}$ (pGlc'$_{50}$Gal'$_{50}$) ^{1}H NMR (500 MHz, $CDCl_3$) δ 7.33 (br, s), 5.87 (br, s), 5.39 (br, s), 5.30 (br, s), 5.21 (br, s), 5.18 (br, s), 5.16 (br, s), 5.08 (br, s), 5.04 (br, s), 4.97 (br, s), 4.52 (br, s), 4.26 (br, s), 4.14 (br, s), 3.95 (br, s), 3.83 (br, s), 3.73 (br, s), 3.67 (br, s), 3.50 (br, s), 3.30 (br, s), 3.02 (br, s), 2.68 (br, s), 2.25 (br, s), 2.16 (s), 2.08 (br, s), 2.04 (br, s), 2.03 (br, s), 2.00 (br, s), 1.99 (br, s), 1.57 (br, s), 1.12 (br, s). ^{13}C NMR (126 MHz, $CDCl_3$) δ 170.58 170.35 170.17 170.04 169.45 101.35 100.86 72.71 71.86 71.31 70.78 69.13 68.27 67.02 61.88 61.26 39.14 20.93 20.81 20.72 20.61. Yield: 66%

Poly(**1a'**)$_{50}$-*ran*-poly(**1c'**)$_{50}$ (pGlc'$_{50}$Fuc'$_{50}$) ^{1}H NMR (500 MHz, $CDCl_3$) δ 7.32 (br, s), 5.87 (br, s), 5.31 (br, s), 5.27 (br, s), 5.19 (br, s), 5.17 (br, s), 5.12 (br, s), 5.06 (br, s), 5.03 (br, s), 4.95 (br, s), 4.52 (br, s), 4.25 (br, s), 4.12 (br, s), 3.81 (br, s), 3.72 (br, s), 3.51 (br, s), 3.33 (br, s), 3.01 (br, s), 2.68 (br, s), 2.24 (br, s), 2.16 (br, s), 2.07 (br, s), 2.06 (br, s), 2.02 (br, s), 1.99 (br, s), 1.98 (br, s), 1.85 (br, s), 1.57 (br, s), 1.12 (br, s). ^{13}C NMR (126 MHz, $CDCl_3$) δ 170.61 170.16 169.45 100.85 96.45 72.73 71.90 71.02 68.29 68.11 67.94 64.59 61.84 39.11 20.81 20.75 20.68 20.62 15.94. Yield: 46.0%

Poly(**1b'**)$_{15}$-*ran*-poly(**1c'**)$_{15}$ (pGal'$_{15}$Fuc'$_{15}$) ^{1}H NMR (500 MHz, $CDCl_3$) δ 7.34 (d), 5.88 (br, s), 5.40 (br, s), 5.32 (br, s), 5.29 (br, s), 5.17 (br, s), 5.15 (br, s), 5.04 (br, s), 4.51 (br, *s*), 4.14 (br, s), 3.96 (br, s), 3.86 (br, s), 3.71 (t), 3.53 (br, s), 3.34 (br, s), 3.04 (br, s), 2.70 (br, s), 2.28 (br, m), 2.17 (br, s), 2.07 (br, s), 2.06 (br, s), 2.00 (br, s), 1.93 (m), 1.59 (br, s), 1.14 (br, s). ^{13}C NMR (126 MHz, $CDCl_3$) δ 170.61 170.36 170.17 170.05 169.58 101.31 96.44 71.03 70.76 68.92 68.12 67.93 67.43 67.01 64.61 61.21 39.12 33.29 20.93 20.70 20.67 20.59 15.94. Yield: 62-74%.

Poly(**1b’**)$_{15}$-*ran*-poly(**1c’**)$_{15}$-*ran*-poly(**1a’**)$_{70}$ (pGal’$_{15}$Fuc’$_{15}$Glc’$_{70}$) ^{1}H NMR (700 MHz, $CDCl_3$) δ 6.00 (br, s), 5.42 (br, s), 5.33 (br, s), 5.30 (br, s), 5.22 (br, s), 5.09 (br, s), 5.05 (br, s), 4.98 (br, s), 4.56 (br, s), 4.29 (br, s), 4.16 (br, s), 3.98 (br, s), 3.84 (br, s), 3.75 (br, s), 3.70 (br, s), 3.54 (br, s), 3.34 (br, s), 3.05 (br, s), 2.70 (br, s), 2.30 (br, s), 2.19 (br, s), 2.11 (br, s), 2.07 (br, s), 2.05 (br, s), 2.03 (br, s), 2.01 (br, s), 1.60 (br, s), 1.15 (br, s). ^{13}C NMR (176 MHz, $CDCl_3$) δ 170.62, 170.16, 169.46, 100.90, 72.70, 71.86, 71.33, 68.28, 61.85, 39.20, 20.81, 20.62. Yield: 33%.

Poly(**1b’**)$_{25}$-*ran*-poly(**1c’**)$_{75}$ (pGal’$_{25}$Fuc’$_{75}$) ^{1}H NMR (700 MHz, $CDCl_3$) δ 6.03 (br, s), 5.41 (br, s), 5.33 (br, s), 5.29 (br, s), 5.16 (br, s), 5.04 (br, s), 4.52 (br, s), 4.15 (br, s), 3.97 (br, s), 3.87 (br, s), 3.74 (br, s), 3.54 (br, s), 3.38 (br, s), 3.06 (br, s), 2.72 (br, s), 2.30 (br, s), 2.18 (br, s), 2.07 (br, s), 2.00 (br, s), 1.67 (br, s), 1.61 (br, s), 1.15 (br, s). ^{13}C NMR (176 MHz, $CDCl_3$) δ 170.64, 170.18, 96.45, 71.04, 70.79, 68.13, 67.94, 64.61, 39.13, 20.92, 20.75, 20.68, 20.60, 15.94. Yield: 40%

Poly(**1b’**)$_{50}$-*ran*-poly(**1c’**)$_{50}$ (pGal’$_{50}$Fuc’$_{50}$) ^{1}H NMR (500 MHz, $CDCl_3$) δ 7.34 (br, d), 5.91 (br, s), 5.40 (br, s), 5.33 (br, s), 5.29 (br, s), 5.18 (br, s), 5.15 (br, s), 5.04 (br, s), 4.51 (br, s), 4.14 (br, s), 3.96 (br, s), 3.86 (br, s), 3.75 (br, s), 3.67 (br, s), 3.53 (br, s), 3.34 (br, s), 3.04 (br, s), 2.70 (br, s), 2.28 (br, s), 2.17 (s), 2.07 (s), 2.06 (s), 2.00 (br, s), 1.86 (m), 1.59 (br, s), 1.14 (br, s). ^{13}C NMR (126 MHz, $CDCl_3$) δ 170.63 170.39 170.19 170.07 101.33 96.44 71.04 70.78 68.13 67.98 67.00 64.58 61.22 39.16 25.62 20.93 20.72 20.68 20.60 15.94. Yield: 38-94%.

Poly(**1b’**)$_{75}$-*ran*-poly(**1c’**)$_{25}$ (pGal’$_{75}$Fuc’$_{25}$) ^{1}H NMR (700 MHz, $CDCl_3$) δ 5.92 (br, s), 5.33 (br, s), 5.25 (br, s), 5.21 (br, s), 5.09 (br, s), 4.96 (br, s), 4.44 (br, s), 4.07 (br, s), 3.89 (br, s), 3.78 (br, s), 3.60 (br, s), 3.46 (br, s), 3.27 (br, s), 2.98 (br, s), 2.63 (br, s), 2.32 (br, s), 2.22 (br, s), 2.09 (br, s), 1.98 (br, s), 1.92 (br, s), 1.52 (br, s), 1.06 (br, s). ^{13}C NMR (176 MHz, $CDCl_3$) δ 170.38, 170.19, 170.07, 101.31, 70.76, 68.98, 68.12, 67.92, 67.02, 61.22, 39.22, 20.94, 20.73, 20.60. Yield: 39%

General deprotection method

The general method of deacetylation was as follows,[1] the protected polymer (10 mg) was dissolved in 2 mL MeOH/THF (2:1, v/v) and to this solution was added K_2CO_3 (75 mg) and the reaction stirred for 20–30 min. The solvents were evaporated, and the solid was then poured into a solution of 10 mL THF/H_2O (1:1, v/v) containing 1N HCl. This solution was then allowed to stir for 30–60 min and the solvents removed in vacuo, followed by ion exchange chromatography for 10-mers or dialysis for 100-mers to afford the deprotected polymer as a white powder.

Poly(**1a**)$_{50}$-*ran*-poly(**1b**)$_{50}$ (pGlc$_{50}$Gal$_{50}$) ^{1}H NMR (500 MHz, D_2O) δ 5.44 (s, 1H), 5.36 (s, 1H), 5.27 (s, 1H), 4.44 (s, 1H), 4.38 (s, 1H), 3.93 (d, J = 16.6 Hz, 1H), 3.77 (d, J = 4.6 Hz, 1H), 3.66 (d, J = 11.5 Hz, 1H), 3.56 (s, 1H), 3.51 (s, 1H), 3.42 (s, 1H), 3.31 (s, 1H), 3.03 (s, 1H), 2.63 (s, 1H), 2.04 (s, 1H), 1.68 (s, 1H). Yield: 54%

Poly(**1a**)$_{50}$-*ran*-poly(**1c**)$_{50}$ (pGlc$_{50}$Fuc$_{50}$) ^{1}H NMR (500 MHz, D_2O) δ 5.43 (br, s), 5.36 (br, s), 4.88 (br, s), 4.45 (br, s), 3.85 (br, s), 3.79 (br, s), 3.75 (br, s), 3.51 (br, s), 3.43 (br, s), 3.31 (br, s), 3.05 (br, s), 2.03 (br, s), 1.68(br, s), 1.23 (br, s). ^{13}C NMR (126 MHz, D_2O) δ 102.46 98.47 75.99 75.73 73.15 71.79 69.64 68.03 66.58 60.84 39.15 15.62. Yield: 95%

Poly(**1b**)$_{15}$-*ran*-poly(**1c**)$_{15}$ (pGal$_{15}$Fuc$_{15}$) ^{1}H NMR (500 MHz, D_2O) δ 5.44 (br, s), 5.36 (br, s), 5.29 (br, s), 4.89 (br, s), 4.39 (br, s), 3.95 (br, s), 3.85 (br, s), 3.79 (br, s), 3.68 (br, s), 3.56 (br, s), 3.44 (br, s), 3.05 (br, s), 2.74 (br, s), 2.52 (br, s), 2.04 (br, s), 1.69 (br, s), 1.23 (br, s). Yield: 62-74%.

Poly(**1b**)$_{15}$-*ran*-poly(**1c**)$_{15}$-*ran*-poly(**1a**)$_{70}$ (pGal$_{15}$Fuc$_{15}$Glc$_{70}$) ^{1}H NMR (700 MHz, $CDCl_3$) δ 5.42 (br, s), 5.34 (br, s), 5.26 (br, s), 4.87 (br, s), 4.43 (br, s), 3.91 (br, s), 3.77 (br, s), 3.73 (br, s), 3.49 (br, s), 3.43 (br, s), 3.40 (br, s), 3.30 (br, s), 3.03 (br, s), 2.75 (br, s), 2.50 (br, s), 2.02 (br, s), 1.84 (br, s), 1.66 (br, s), 1.21 (br, s). Yield: 56%

Poly(**1b**)$_{25}$-*ran*-poly(**1c**)$_{75}$ (pGal$_{25}$Fuc$_{75}$) ^{1}H NMR (700 MHz, $CDCl_3$) δ 5.42 (br, s), 5.34 (br, s), 5.26 (br, s), 4.38 (br, s), 3.99 (br, s), 3.94 (br, s), 3.85 (br, s), 3.78 (br, s), 3.70 (br, s), 3.65 (br, s), 3.56 (br, s), 3.51 (br, s), 3.45 (br, s), 3.37 (br, s), 3.04 (br, s), 2.96 (br, s), 2.75 (br, s), 2.50 (br, s), 2.04 (br, s), 1.66 (br, s), 1.22 (br, s). Yield: 78%

Poly(**1b**)$_{50}$-*ran*-poly(**1c**)$_{50}$ (pGal$_{50}$Fuc$_{50}$) ^{1}H NMR (700 MHz, D_2O) δ 7.30 (br, s), 7.21 (br, s), 5.31 (br, s), 5.24 (br, s), 5.15 (br, s), 4.77 (br, s), 4.27 (br, s), 3.88 (br, s), 3.83 (s), 3.74 (br, s), 3.67 (s), 3.55 (br, s), 3.54 (br, s), 3.45 (br, s), 3.40 (br, s), 3.31 (br, s), 3.24 (br, s), 2.93 (br, s), 2.84 (br, s), 2.64 (br, s), 2.51 (br, s), 2.40 (br, s), 1.92 (br, s), 1.75 (br, s), 1.57 (br, s). Yield: 31-70%

Poly(**1b**)$_{75}$-*ran*-poly(**1c**)$_{25}$ (pGal$_{75}$Fuc$_{25}$) ^{1}H NMR (700 MHz, $CDCl_3$) δ 5.44 (br, s), 5.36 (br, s), 5.28 (br, s), 4.89 (br, s), 4.39 (br, s), 3.95 (br, s), 3.85 (br, s), 3.79 (br, s), 3.66 (br, s), 3.56 (br, s), 3.44 (br, s), 3.04 (br, s), 2.77 (br, s), 2.52 (br, s), 2.04 (br, s), 1.87 (br, s), 1.69 (br, s), 1.23 (br, s). Yield: 74%.

Peacetylated glycopolymers

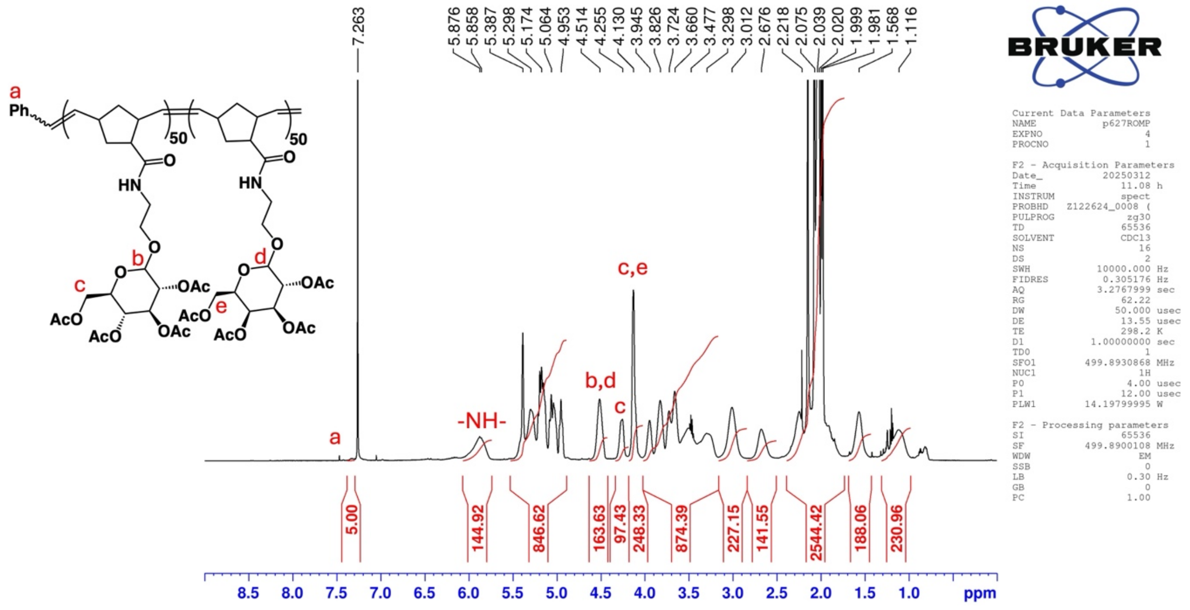

Figure S1. ^{1}H-NMR of poly(**1a'**)$_{50}$-*ran*-(**1b'**)$_{50}$ ^{1}H-NMR using styrene end-group analysis: the aromatic protons (5H) at δ 7.30 ppm determines the proton integration of the anomeric carbon for each glycomonomer, the 1a':1b' ratio = 1.4 : 1.

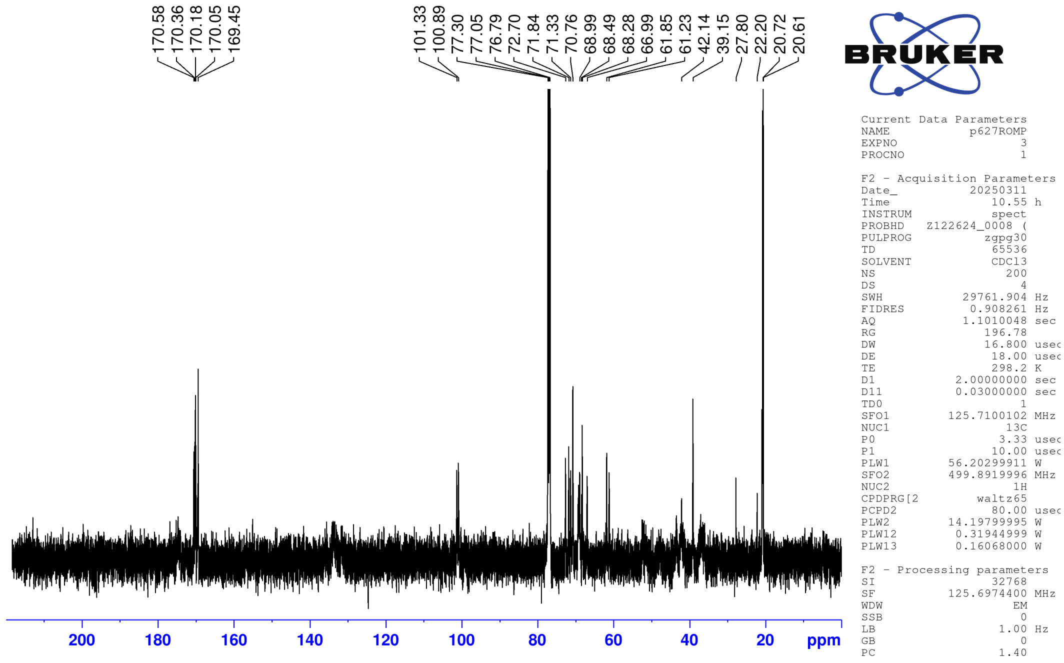

Figure S2 ^{13}C-NMR of poly(**1a'**)$_{50}$-*ran*-(**1b'**)$_{50}$

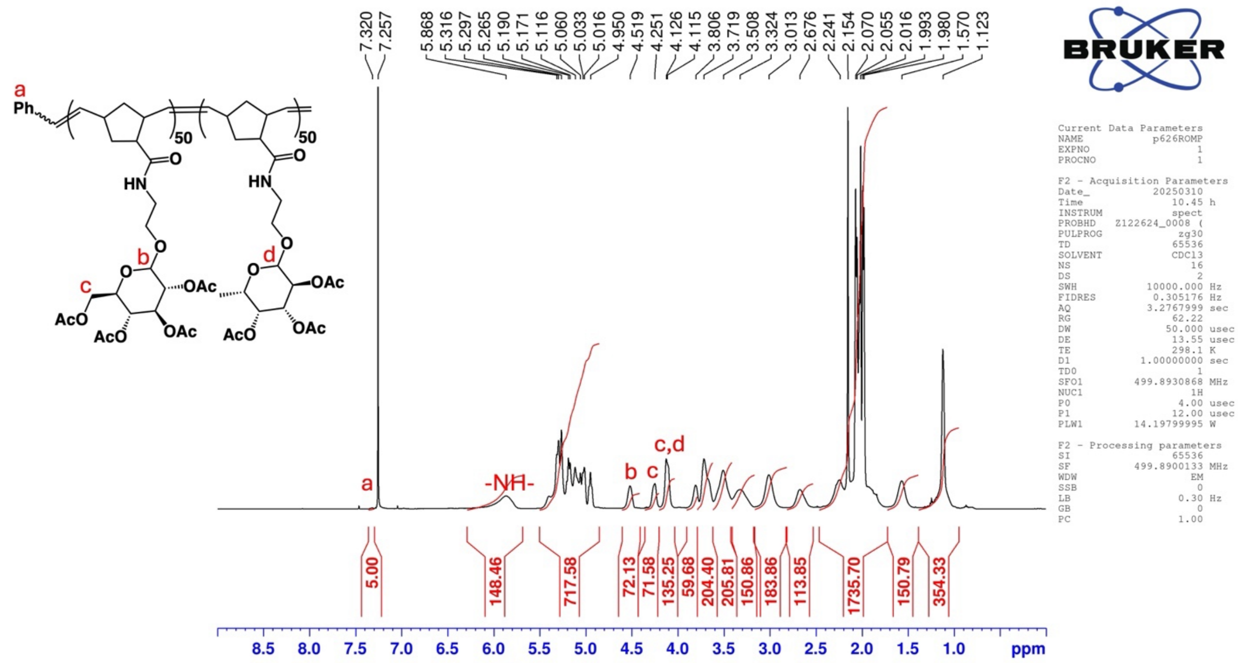

Figure S3. ^{1}H-NMR of poly(**1a’**)$_{50}$-*ran*-(**1c’**)$_{50}$ ^{1}H-NMR using styrene end-group analysis: the aromatic protons (5H) at δ 7.30 ppm determines the proton integration of the anomeric carbon for each glycomonomer, the 1a’:1b’ ratio = 1.1:1.

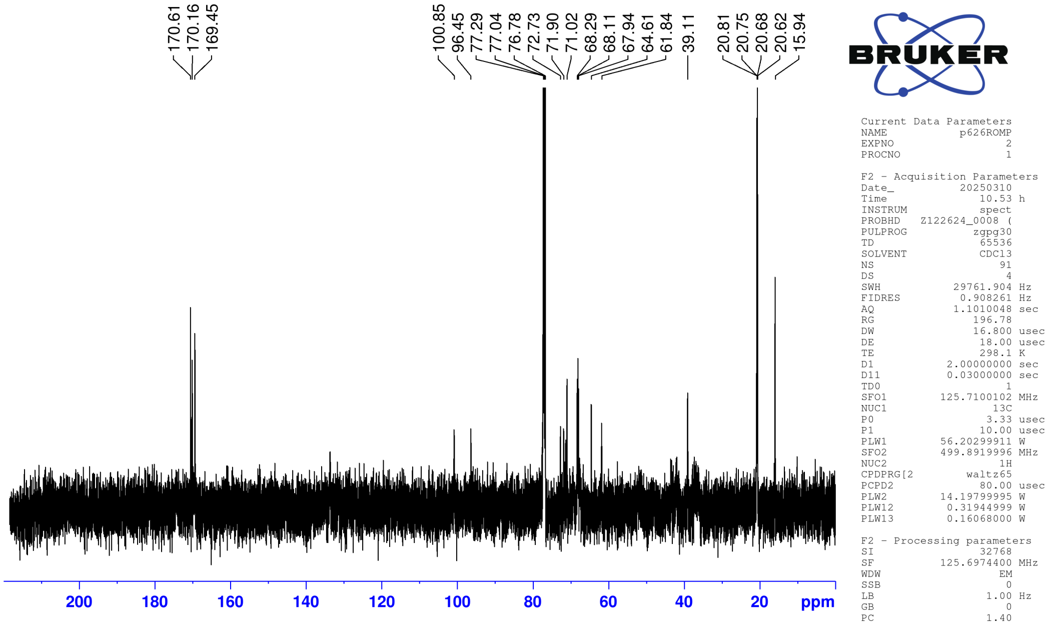

Figure S4. ^{13}C-NMR of poly(**1a’**)$_{50}$-*ran*-(**1c’**)$_{50}$

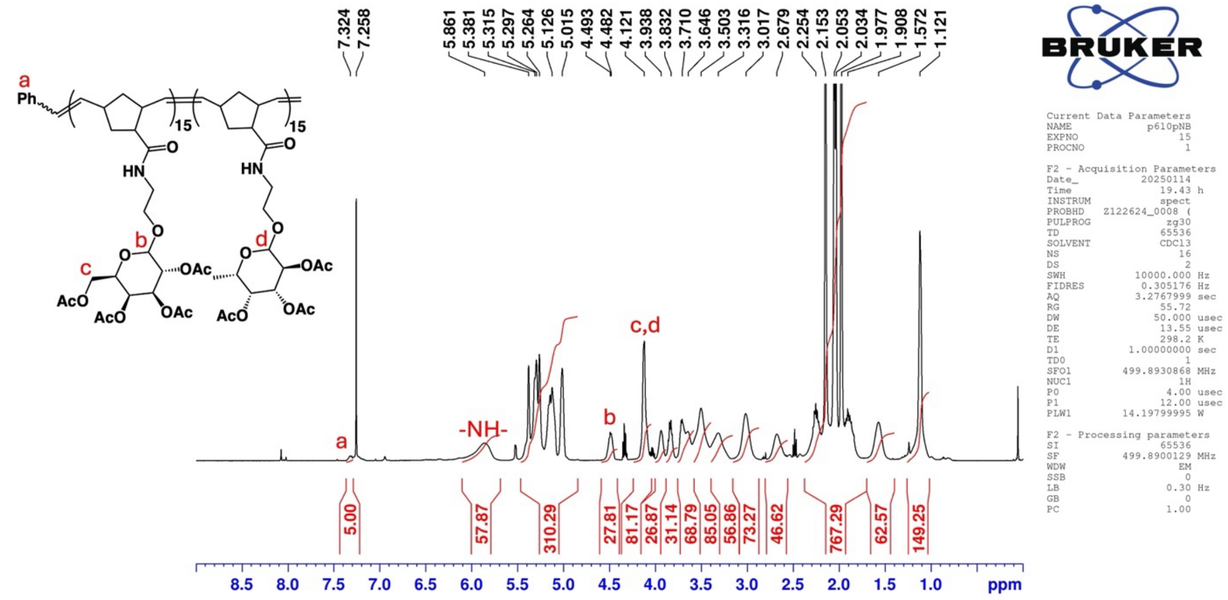

Figure S5. ^{1}H-NMR of poly(**1b'**)$_{15}$-*ran*-(**1c'**)$_{15}$ ^{1}H-NMR using styrene end-group analysis: the aromatic protons (5H) at δ 7.30 ppm determines the proton integration of the anomeric carbon for each glycomonomer, the 1b':1c' ratio = 1:1.2.

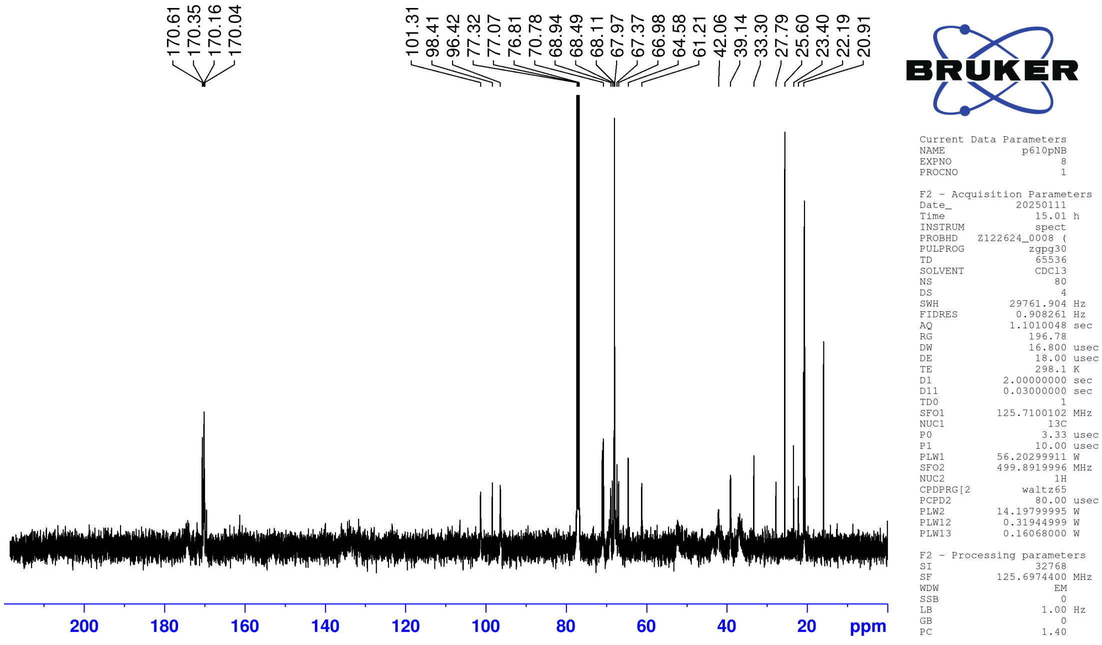

Figure S6. ^{13}C-NMR of poly(**1b'**)$_{15}$-*ran*-(**1c'**)$_{15}$

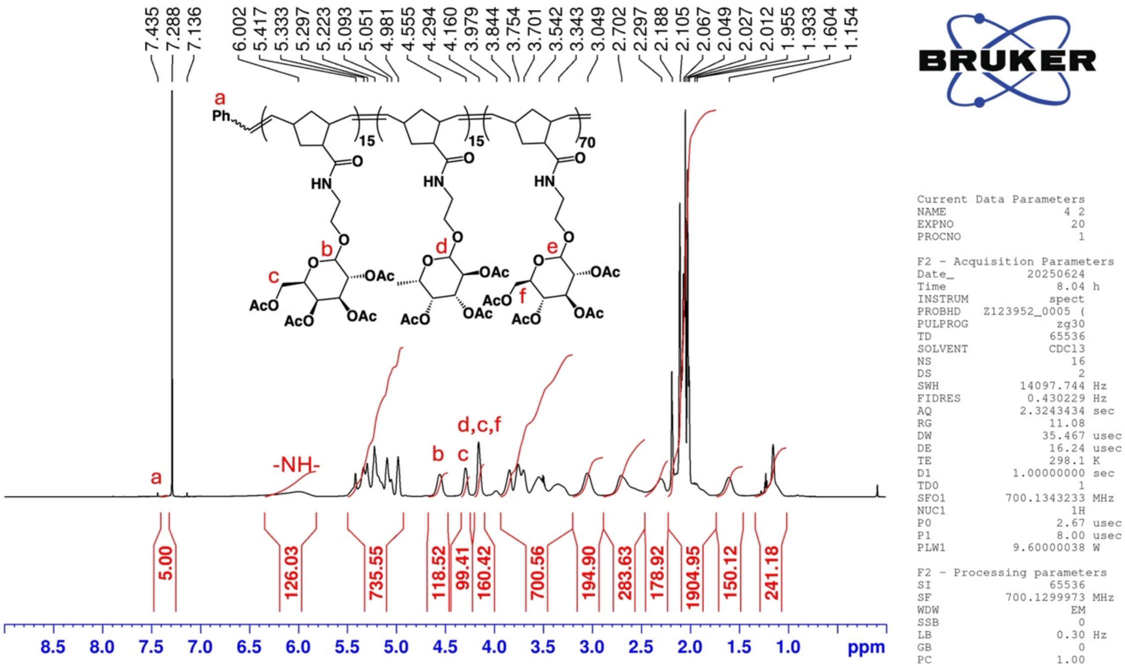

Figure S7. ^{1}H-NMR of poly(**1b'**)$_{15}$-*ran*-(**1c'**)$_{15}$-*ran*-(**1a'**)$_{70}$ ^{1}H-NMR using styrene end-group analysis: the aromatic protons (5H) at δ 7.30 ppm determines the proton integration of the anomeric carbon for each glycomonomer, the 1b':1c':1a' ratio = 1:1.2:5.2.

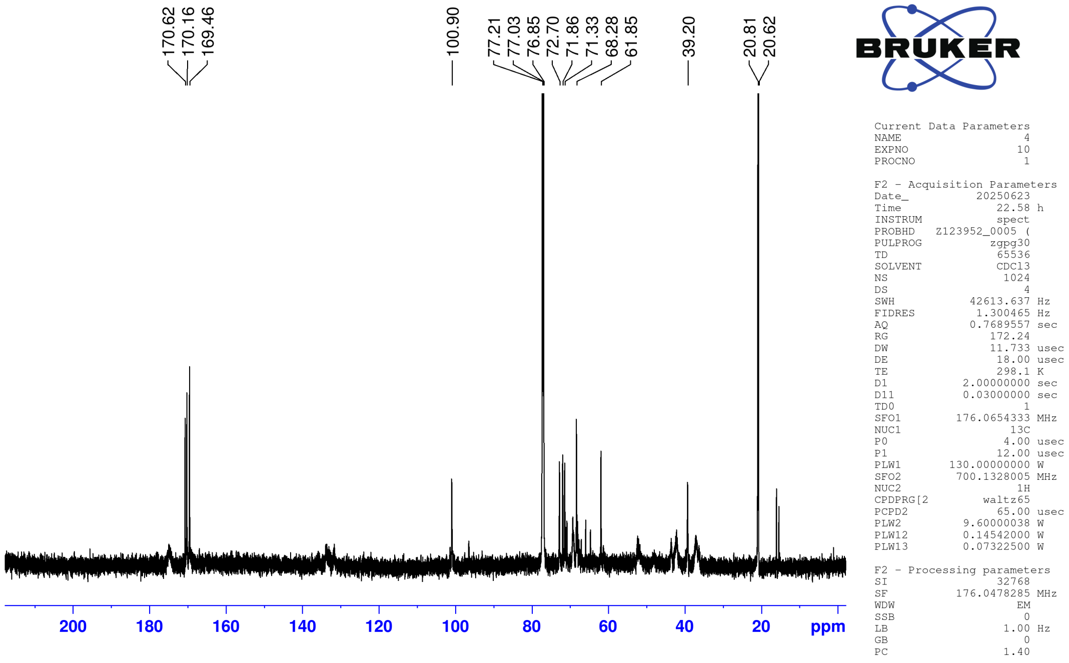

Figure S8. ^{13}C-NMR of poly(**1b'**)$_{15}$-*ran*-(**1c'**)$_{15}$-*ran-(***1a'**)$_{70}$

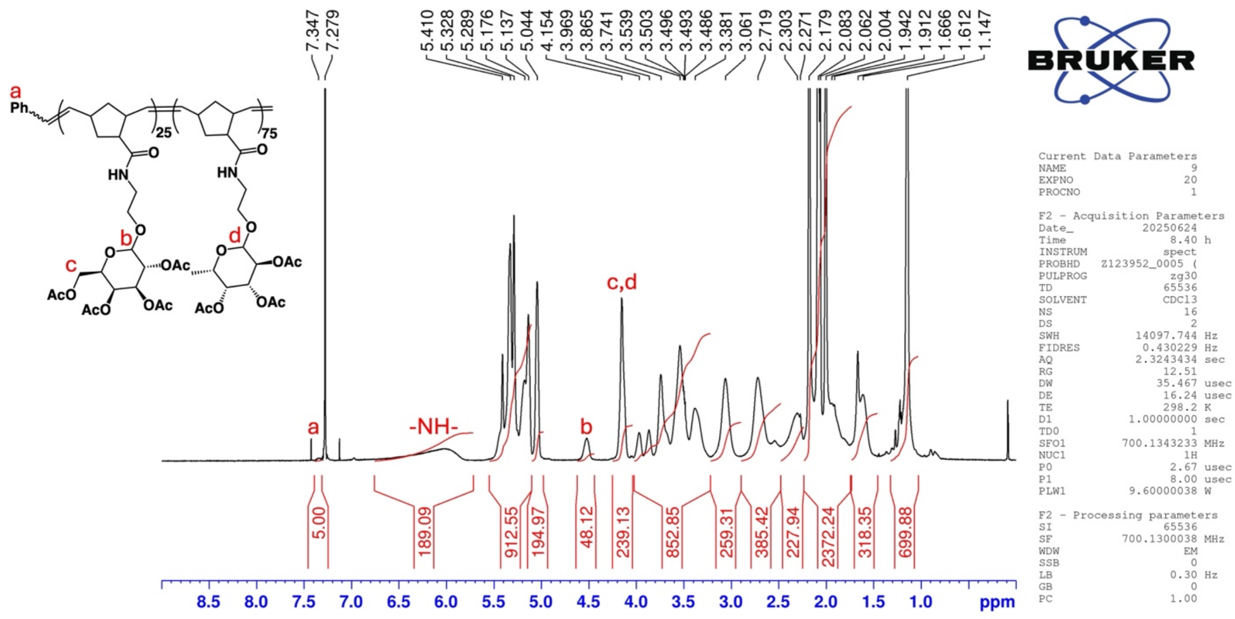

Figure S9. ^{1}H-NMR of poly(**1b’**)$_{25}$-*ran*-(**1c’**)$_{75}$ ^{1}H-NMR using styrene end-group analysis: the aromatic protons (5H) at δ 7.30 ppm determines the proton integration of the anomeric carbon for each glycomonomer, the 1b’:1c’ ratio = 1:2.9.

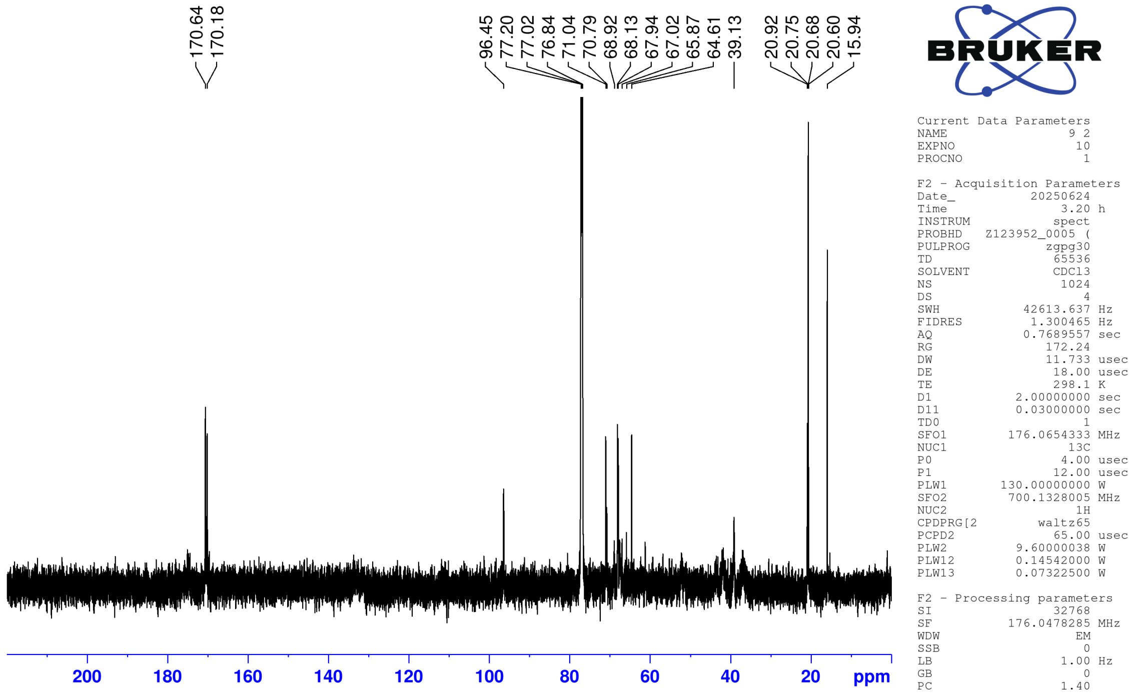

Figure S10. ^{13}C-NMR of poly(**1b’**)$_{25}$-*ran*-(**1c’**)$_{75}$

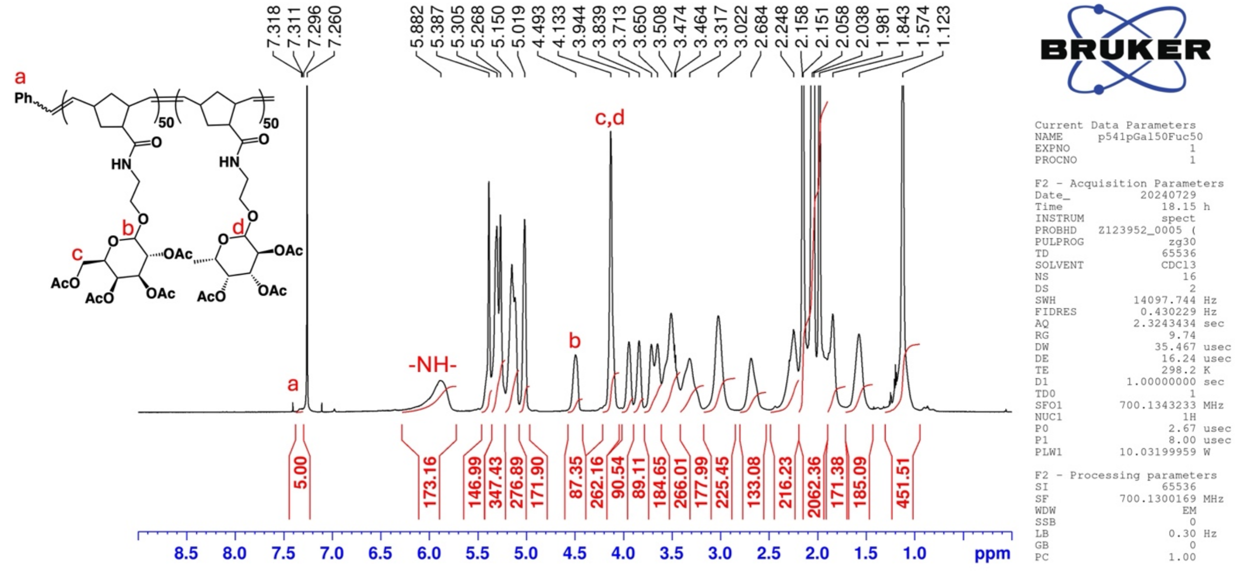

Figure S11. ^{1}H-NMR of poly(**1b'**)$_{50}$-ran-(**1c'**)$_{50}$ ^{1}H-NMR using styrene end-group analysis: the aromatic protons (5H) at δ 7.30 ppm determines the proton integration of the anomeric carbon for each glycomonomer, the 1b':1c' ratio = 1:1.

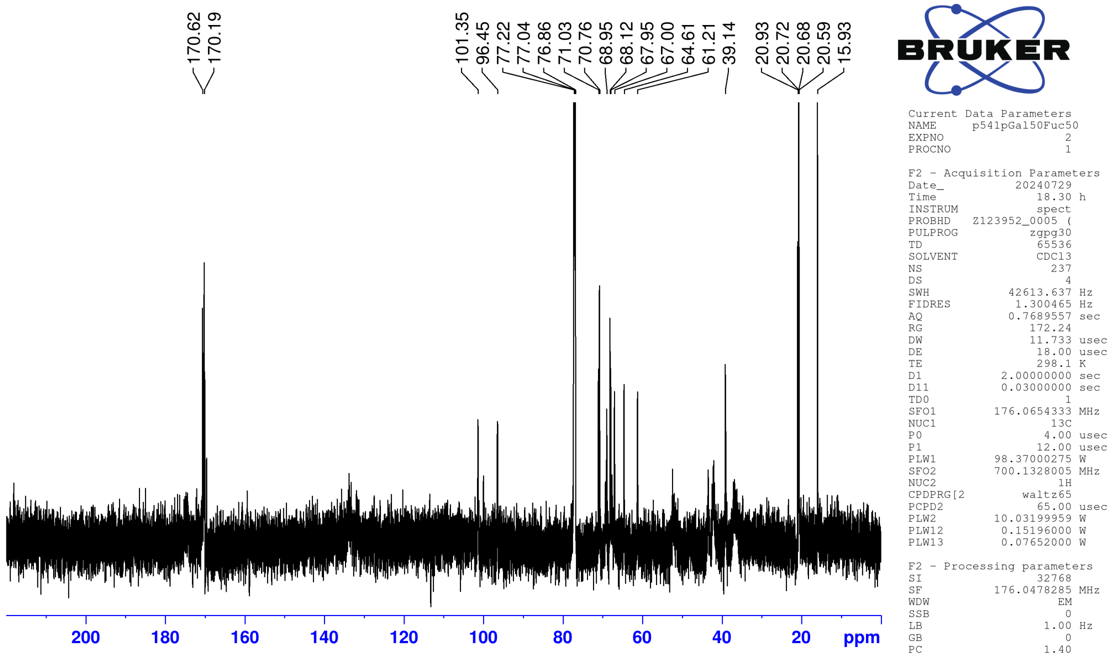

Figure S12. ^{13}C-NMR of poly(**1b'**)$_{50}$-ran-(**1c'**)$_{50}$

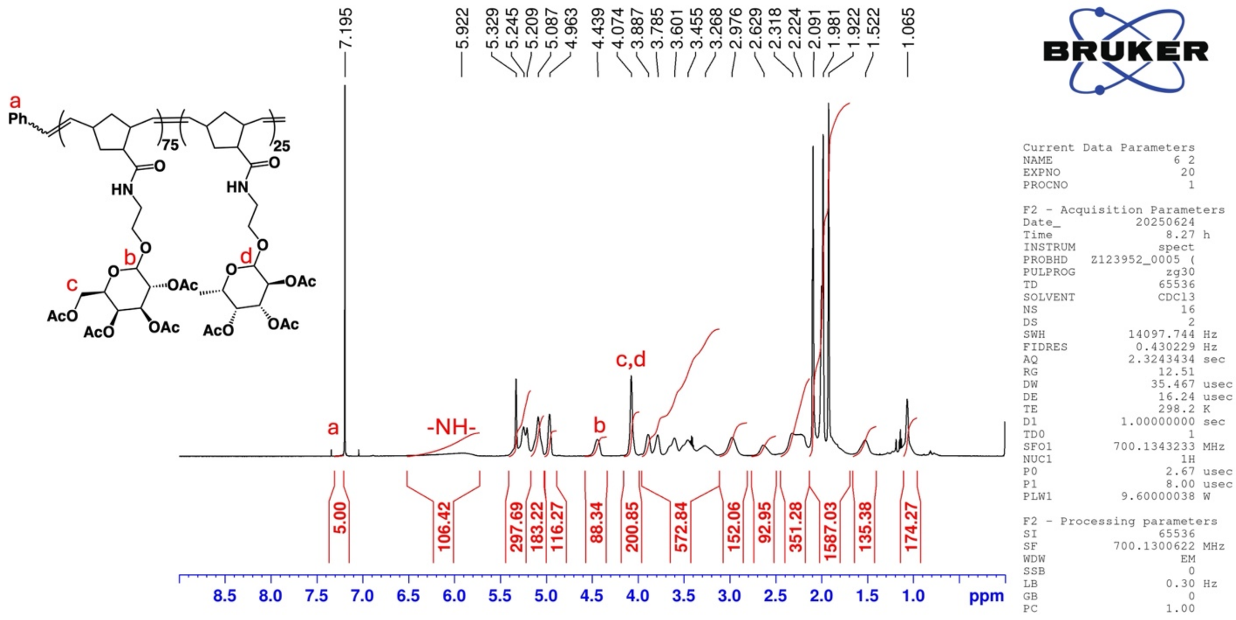

Figure S13.^{1}H-NMR of poly(**1b'**)$_{75}$-*ran*-(**1c'**)$_{25}$ ^{1}H-NMR using styrene end-group analysis: the aromatic protons (5H) at δ 7.30 ppm determines the proton integration of the anomeric carbon for each glycomonomer, the 1b':1c' ratio = 3.6:1.

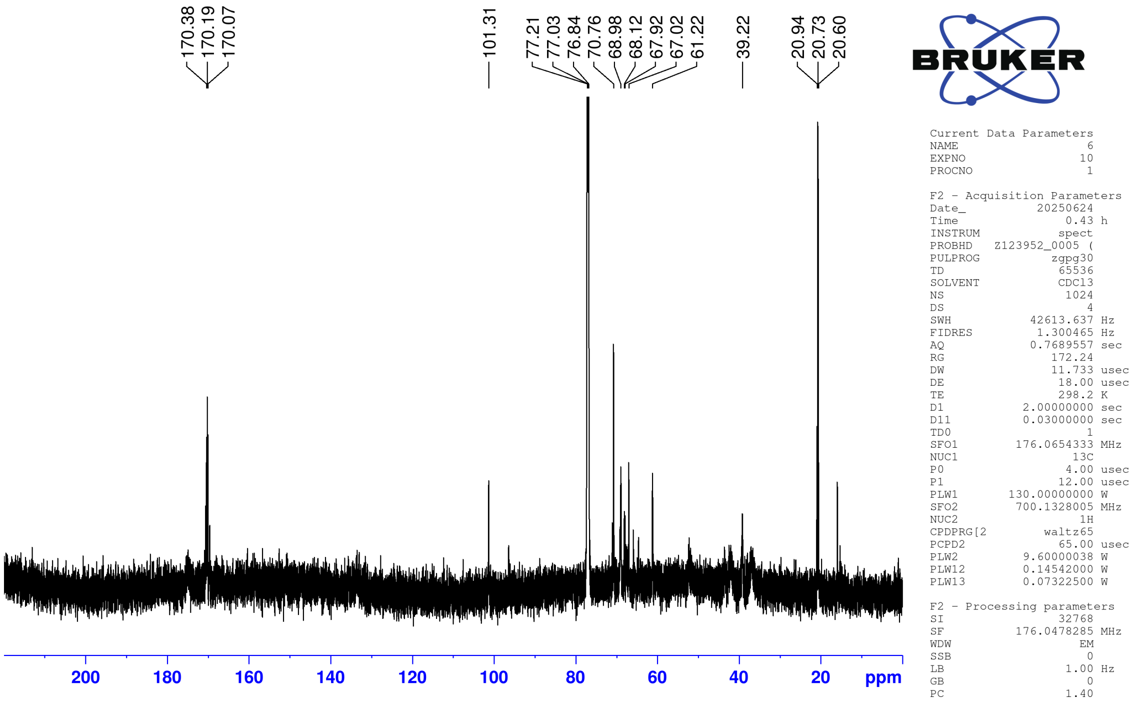

Figure S14. ^{13}C-NMR of poly(**1b'**)$_{75}$-*ran*-(**1c'**)$_{25}$

Deprotected Glycopolymers

^1H NMR (D_2O): Signals at 5.5–5.3 ppm corresponds to olefinic protons of the ROMP backbone. The resonance at ~4.5 ppm, together with an adjacent peak, is assigned to the anomeric proton of the sugar. Broad, overlapping signals between 4.0–3.5 ppm are attributed to sugar ring protons, while resonances at 3.5–3.0 ppm arise from linker and sugar-adjacent methylene protons. Signals in the 2.7–2.0 ppm region are assigned to aliphatic CH and CH_2 protons of the norbornene backbone.

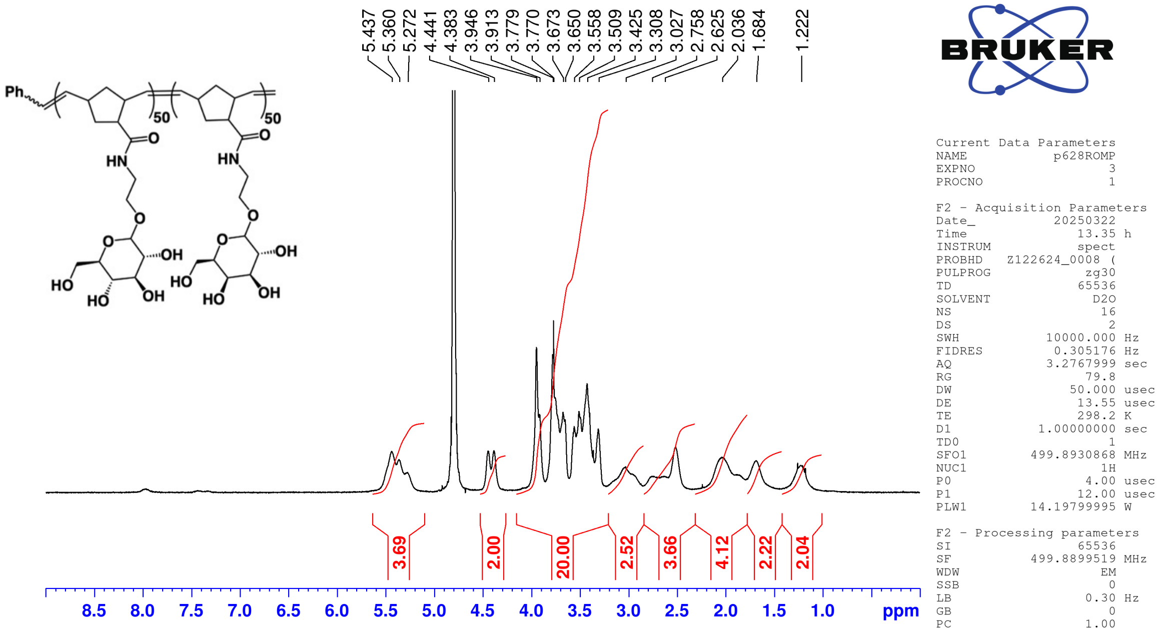

Figure S15. ^{1}H-NMR of poly(**1a**)$_{50}$-*ran*-(**1b**)$_{50}$

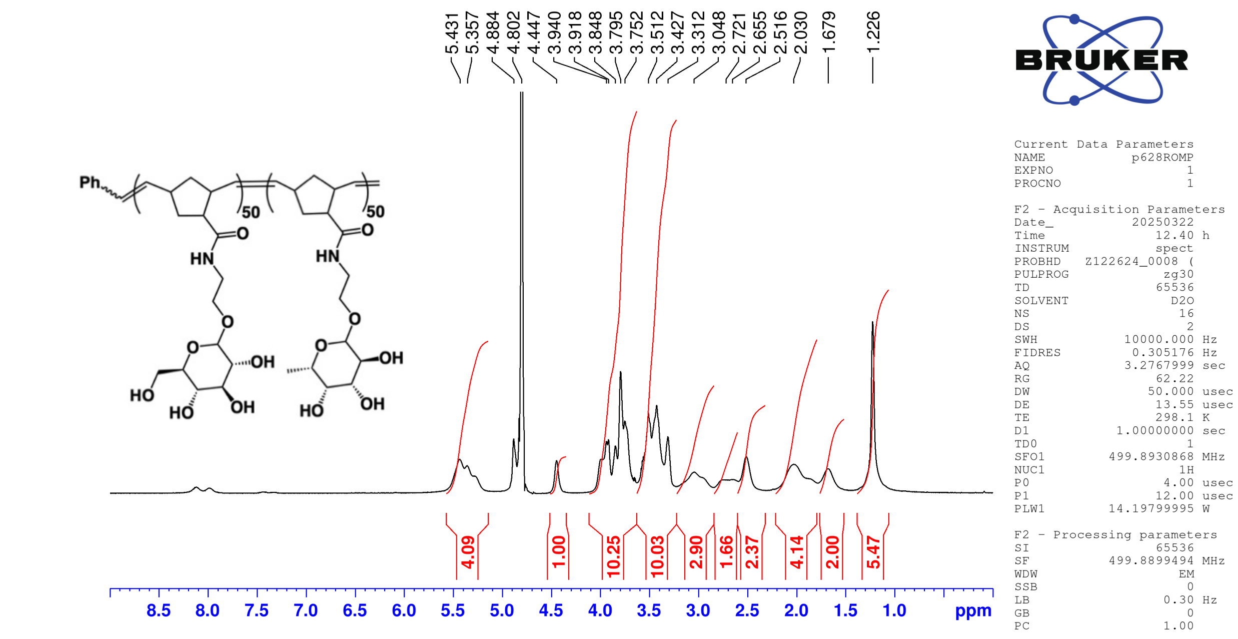

Figure S16. ^{1}H-NMR of poly(**1a**)$_{50}$-*ran*-(**1c**)$_{50}$

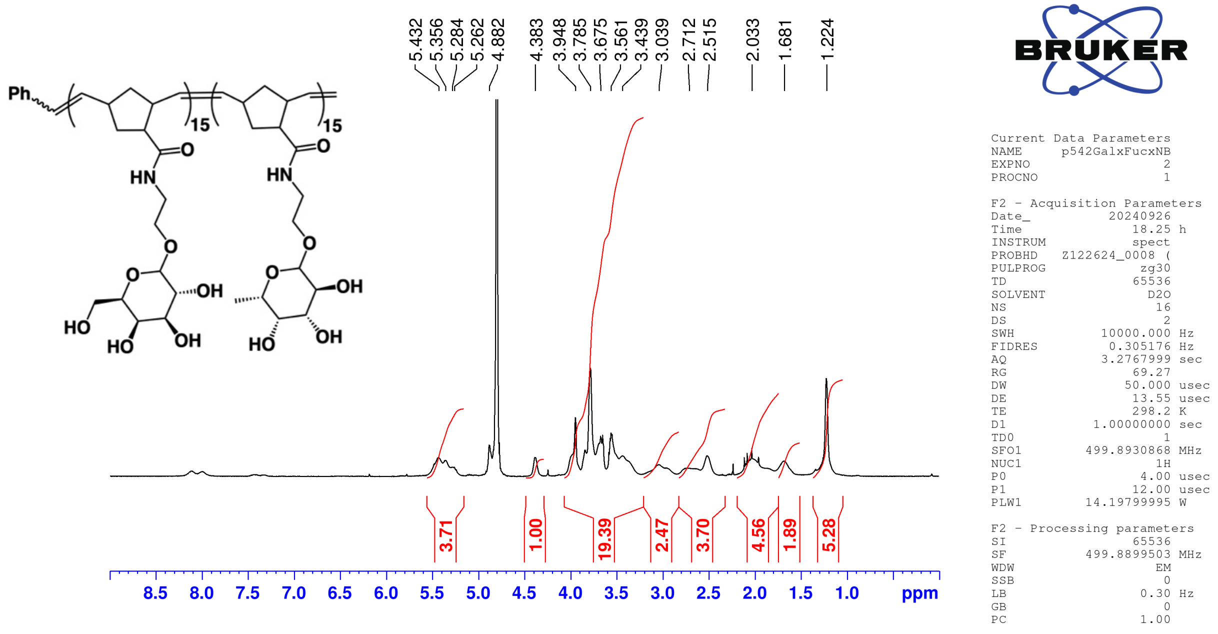

Figure S17. ^{1}H-NMR of poly(**1b**)$_{15}$-*ran*-(**1c**)$_{15}$

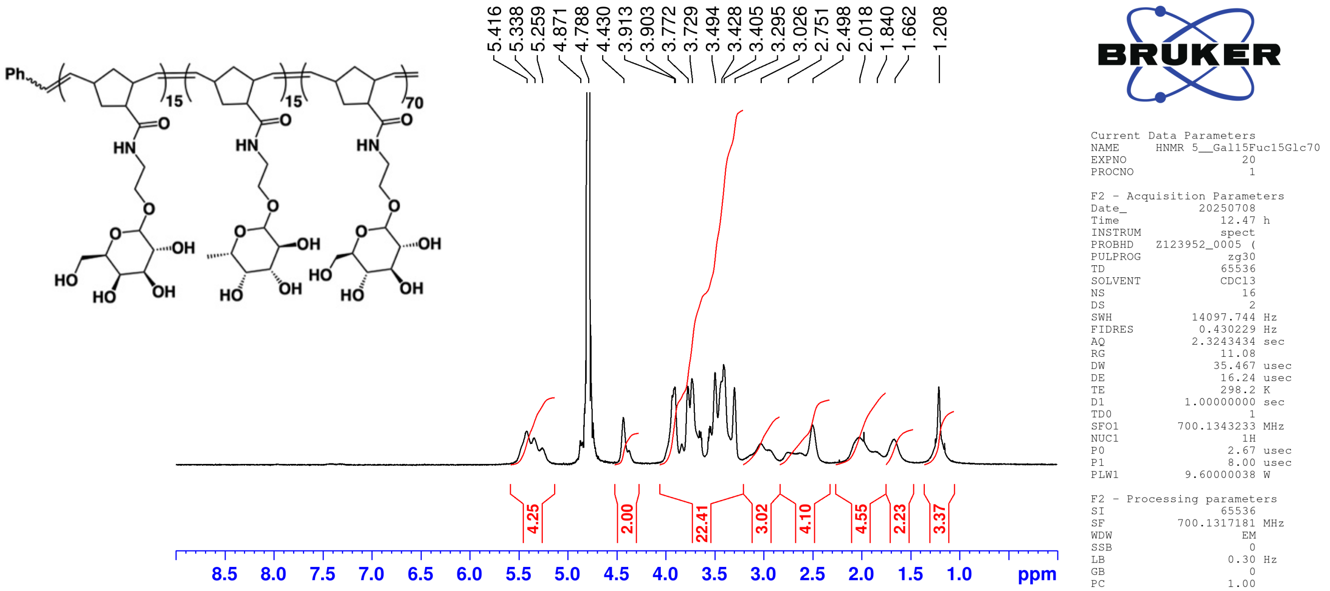

Figure S18. ^{1}H-NMR of poly(**1b**)$_{15}$-*ran*-(**1c**)$_{15}$-*ran*-(**1a**)$_{70}$

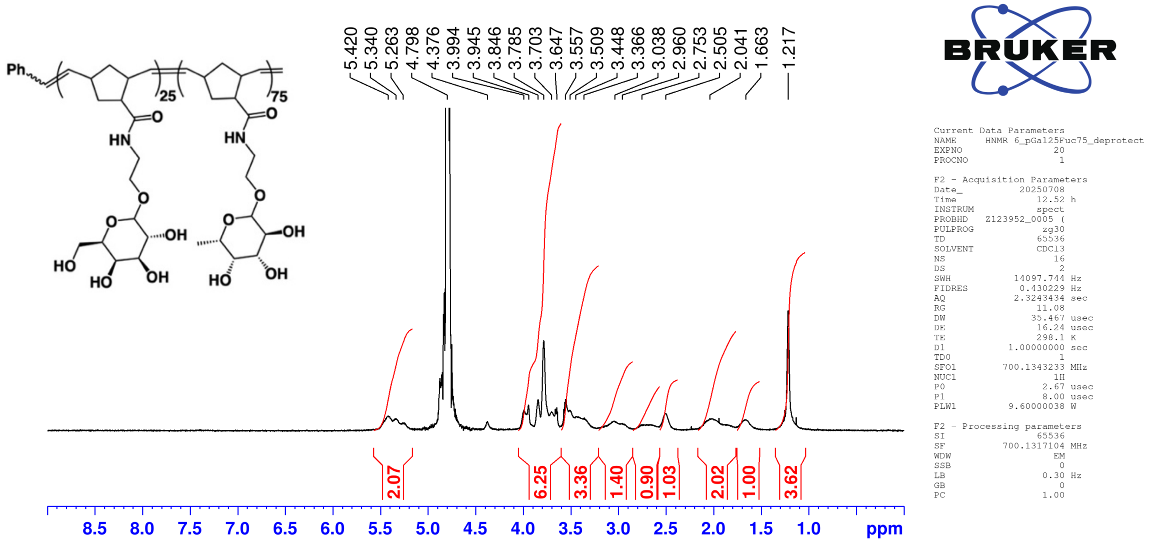

Figure S19. ^{1}H-NMR of poly(**1b**)$_{25}$-*ran*-(**1c**)$_{75}$

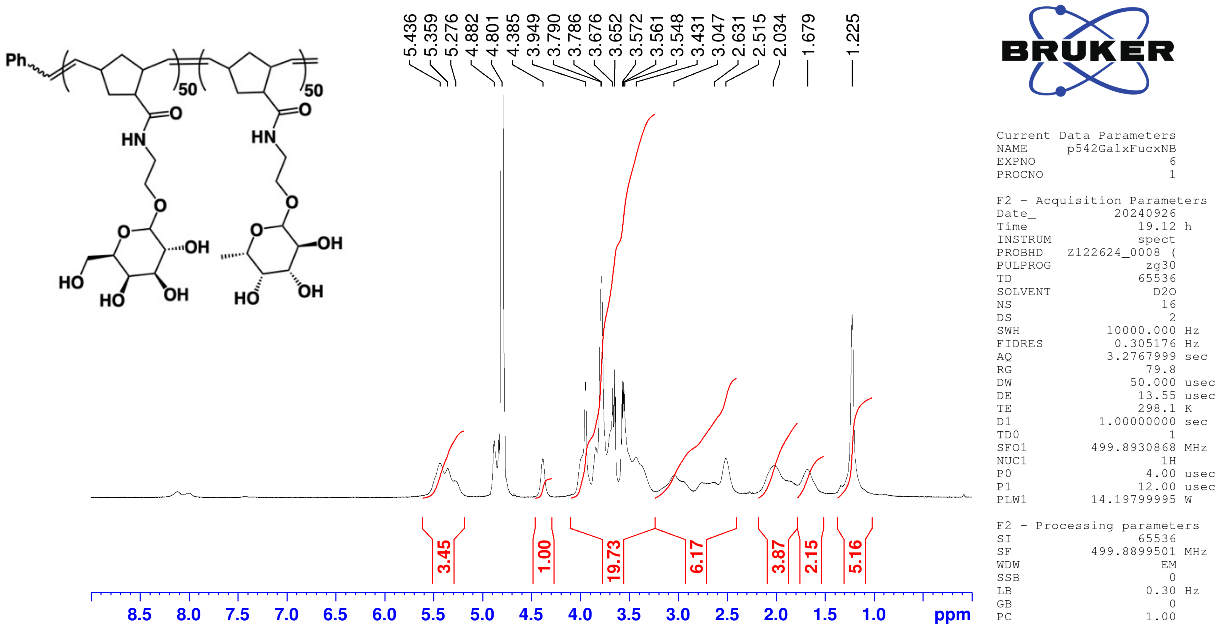

Figure S20. ^{1}H-NMR of poly(**1b**)$_{50}$-*ran*-(**1c**)$_{50}$

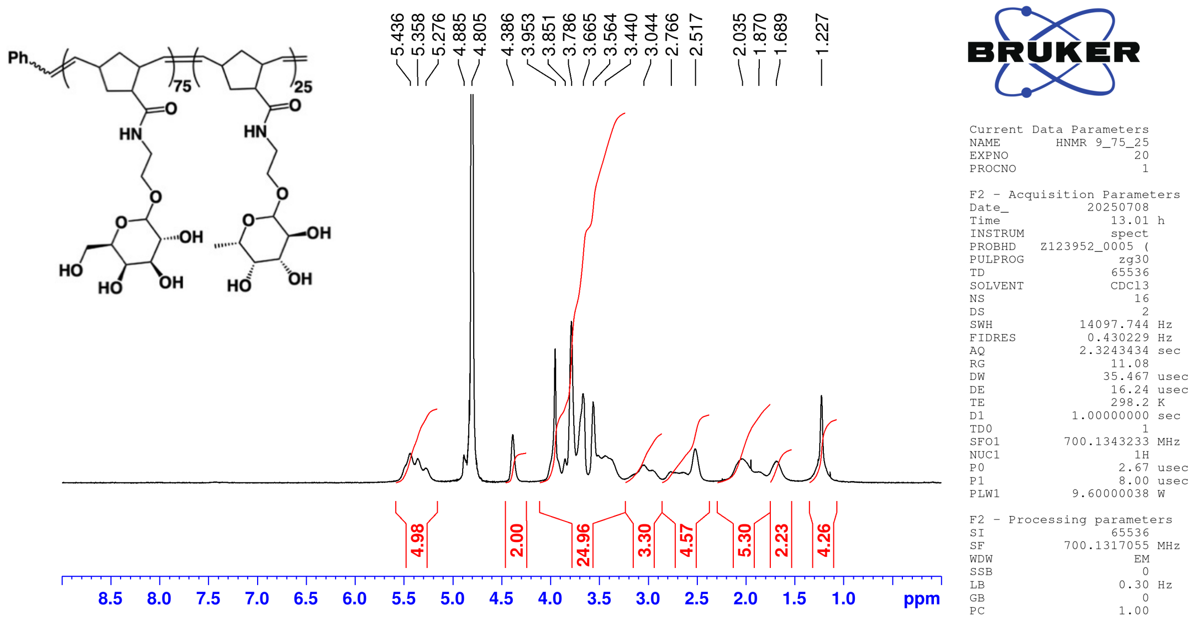

Figure S21.^{1}H-NMR of poly(**1b**)$_{75}$-*ran*-(**1c**)$_{25}$

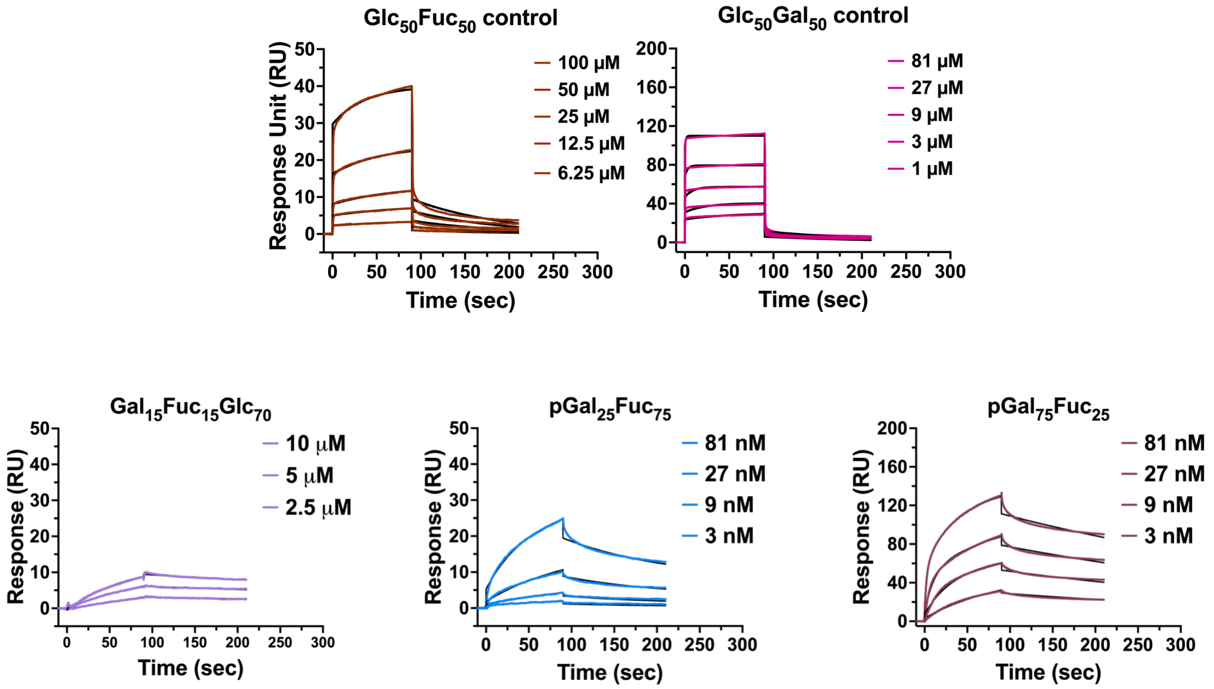

Figure S22. SPR sensorgrams of copolymers with (1) upper row: glucose controls poly(**1a**)$_{50}$-*ran*-poly(**1c**)$_{50}$ (pGlc$_{50}$Fuc$_{50}$) and poly(**1a**)$_{50}$-*ran*-poly(**1b**)$_{50}$ (pGlc$_{50}$Gal$_{50}$); (2) Bottom row: poly(**1b**)$_{15}$-*ran*-poly(**1c**)$_{15}$-*ran*-poly(**1a**)$_{70}$ (pGal$_{15}$Fuc$_{15}$Glc$_{70}$, left), poly(**1b**)$_{25}$-*ran*-poly(**1c**)$_{75}$ (pGal$_{25}$Fuc$_{75}$, middle), poly(**1b**)$_{75}$-*ran*-poly(**1c**)$_{25}$ (pGal$_{75}$Fuc$_{25}$, right).

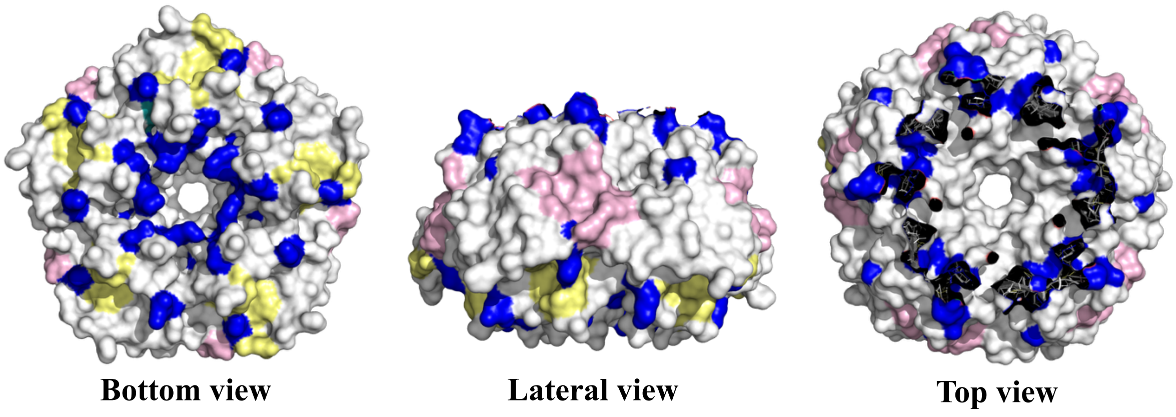

Figure S23. Lysine distribution on the surface of CTB. The CTB structure is based on pdb_00006hmy. Structures are visualized using PyMol.3 Light yellow and pink color the canonical and noncanonical binding sites, respectively. The blue residues are surface lysine.

References

(1) Wu, L.; Sampson, N. S. Fucose, Mannose, and β-N-Acetylglucosamine Glycopolymers Initiate the Mouse Sperm Acrosome Reaction through Convergent Signaling Pathways. *ACS Chem. Biol.* **2014**, *9* (2), 468–475. https://doi.org/10.1021/cb400550j.

(2) Cervin, J.; Boucher, A.; Youn, G.; Björklund, P.; Wallenius, V.; Mottram, L.; Sampson, N. S.; Yrlid, U. Fucose-Galactose Polymers Inhibit Cholera Toxin Binding to Fucosylated Structures and Galactose-Dependent Intoxication of Human Enteroids. *ACS Infect. Dis.* **2020**, *6* (5), 1192–1203. https://doi.org/10.1021/acsinfecdis.0c00009.

(3) Schrödinger, LLC. The PyMOL Molecular Graphics System, Version 1.8, 2015.
